# A matrisome RNA signature from early-pregnancy mouse mammary fibroblasts predicts distant metastasis-free breast cancer survival in humans

**DOI:** 10.1186/s13058-021-01470-3

**Published:** 2021-09-26

**Authors:** Ayman M. Ibrahim, Alan Bilsland, Steffen Rickelt, Joanna S. Morris, Torsten Stein

**Affiliations:** 1 Institute of Cancer Sciences, College of MVLS, University of Glasgow, Glasgow, G12 8QQ UK; 2 Zoology Department, Faculty of Science, Cairo University, Giza, 12613 Egypt; 3 Present Address: Aswan Heart Centre, Aswan, 200 Egypt; 4 Glasgow Experimental Cancer Medicines Centre, Institute of Cancer Science, College of MVLS, University of Glasgow, Glasgow, G12 8QQ UK; 5 David H. Koch Institute for Integrative Cancer Research, MIT, Cambridge, USA; 6 School of Veterinary Medicine, College of MVLS, University of Glasgow, Bearsden Road, Glasgow, G61 1QH UK; 7 School of Medicine, College of MVLS, University of Glasgow, Glasgow, G12 8QQ UK; 8 Present Address: Institute of Veterinary Biochemistry, Freie Universität Berlin, Oertzenweg 19b, 14163 Berlin, Germany

**Keywords:** Mammary gland, Fibroblasts, Branching, Microarray, Extracellular matrix

## Abstract

**Background:**

During pregnancy, the mouse mammary ductal epithelium branches and grows into the surrounding stroma, requiring extensive extracellular matrix (ECM) and tissue remodelling. It therefore shows parallels to cancer invasion. We hypothesised that similar molecular mechanisms may be utilised in both processes, and that assessment of the stromal changes during pregnancy-associated branching may depict the stromal involvement during human breast cancer progression.

**Methods:**

Immunohistochemistry (IHC) was employed to assess the alterations within the mouse mammary gland extracellular matrix during early pregnancy when lateral branching of the primary ductal epithelium is initiated. Primary mouse mammary fibroblasts from three-day pregnant and age-matched non-pregnant control mice, respectively, were 3D co-cultured with mammary epithelial cells to assess differences in their abilities to induce branching morphogenesis in vitro. Transcriptome analysis was performed to identify the underlying molecular changes. A signature of the human orthologues of the differentially expressed matrisome RNAs was analysed by Kaplan–Meier and multi-variate analysis in two large breast cancer RNA datasets (Gene expression-based Outcome for Breast cancer Online (GOBO) und Kaplan–Meier Plotter), respectively, to test for similarities in expression between early-pregnancy mouse mammary gland development and breast cancer progression.

**Results:**

The ECM surrounding the primary ductal network showed significant differences in collagen and basement membrane protein distribution early during pregnancy. Pregnancy-associated fibroblasts (PAFs) significantly enhanced branching initiation compared to age-matched control fibroblast. A combined signature of 64 differentially expressed RNAs, encoding matrisome proteins, was a strong prognostic indicator of distant metastasis-free survival (DMFS) independent of other clinical parameters. The prognostic power could be significantly strengthened by using only a subset of 18 RNAs (LogRank *P* ≤ 1.00e−13; Hazard ratio (HR) = 2.42 (1.8–3.26); *p* = 5.61e−09). The prognostic power was confirmed in a second breast cancer dataset, as well as in datasets from ovarian and lung cancer patients.

**Conclusions:**

Our results describe for the first time the early stromal changes that accompany pregnancy-associated branching morphogenesis in mice, specify the early pregnancy-associated molecular alterations in mouse mammary fibroblasts, and identify a matrisome signature as a strong prognostic indicator of human breast cancer progression, with particular strength in oestrogen receptor (ER)-negative breast cancers.

**Supplementary Information:**

The online version contains supplementary material available at 10.1186/s13058-021-01470-3.

## Background

Stromal-epithelial interactions control epithelial cell growth during normal organ development and cancer progression [1]. Fibroblasts, as a major stromal cell type, play key roles in controlling development and cancer progression-associated histological changes [2]. While normal fibroblasts can suppress tumour growth, cancer-associated fibroblasts (CAFs) have been reported to support or even induce growth, invasion and metastasis [2, 3]. However, CAFs are highly heterogeneous and can even suppress tumour growth [4, 5]. No single biomarker is so far described that would either clearly define CAFs or identify a specific tumour suppressive or supportive role [5]. Their mechanisms of action are accordingly also highly complex, affecting growth factor-induced proliferation, remodelling of ECM and neo-vascularisation [2]. Thus, our understanding of how fibroblasts control cancer progression is still limited. It has therefore been suggested that in order to fully comprehend the role(s) of CAFs during cancer progression one first needs to understand the roles of normal fibroblasts in controlling epithelial cell growth [6].

The developing mouse mammary gland is an excellent model system to study stromal influence on epithelial growth as it develops mainly postnatally, showing significant morphological changes. Furthermore, remodelling processes similar to those seen during breast cancer progression are also observed during normal mammary branching morphogenesis [7, 8]. At puberty, a rudimentary epithelium grows into the surrounding mammary fat pad helped by highly proliferative terminal end buds (TEB), forming a branched primary ductal network behind them [9]. While ECM at the growth front of TEBs mainly contains a thin layer of hyaluronic acid-rich basement membrane (BM), ECM of the neck region is defined by a thick surrounding layer of fibrous collagenous BM/ECM [7, 9], which continues along the developing milk ducts. During pregnancy, these ducts form lateral side branches and alveoli, a process which requires remodelling of the surrounding ECM; specifically, breakdown of existing BM/collagen sheath and formation of a new BM and collagen network [10]. This process could therefore be described as ‘controlled invasion’. Epithelial-stromal interactions are crucial for these morphological changes to occur [1] and fibroblasts play an essential part [10, 11]. However, our understanding of the molecular processes involved and how fibroblasts enable ductal branching remains limited.

By enzymatically isolating TEB and mammary ducts, we previously identified the involvement of axon-guidance proteins [12] as well as the involvement of BM proteins fibulin-2 (FBLN2) and versican (VCAN) in pubertal ductal development [13, 14]. We also recently established a method, which enabled us to carry out whole-genome transcriptome analysis on RNA from very small populations of freshly isolated, non-cultured mammary fibroblasts using linear amplification [15].

Here we used this method to characterise the mammary fibroblast transcriptome of 3 day-pregnant and age-matched control mice. We focussed on RNAs encoding proteins of the matrisome, which comprises core ECM-proteins, ECM-modifying enzymes, growth factors, and matricellular proteins [16]. The identified RNAs describe a network of growth factor activation, protease expression, collagen sheath breakdown, and induced ECM/BM formation, thereby identifying potential new control mechanisms for epithelial outgrowth. Consistent with our hypothesis, this pregnancy-associated RNA signature of matrisome genes was able to significantly predict distant metastasis-free and recurrence-free survival in a dataset of 1881 human breast cancers [17] independent of other clinical parameters, as well as progression-free survival in patients with lung or ovarian cancer. Our data therefore sheds important new light onto potential regulators of breast cancer progression and provides a potential new matrisome-based prognostic marker for the risk of developing distant metastases.

## Material and methods

### Animal husbandry

Mice (strain C57BL/6) were kept in conventional M3 cages bedded with wood chips and paper nesting material in a temperature-controlled environment at 21 ± 1 °C, 45–55% humidity on a 12-h light/dark cycle. Food and water were provided ad libitum. Mice were allowed a 7-day acclimatisation period after arrival on site prior to experimental use.

### Fibroblast enrichment

Primary mammary fibroblast-enriched extracts were isolated as previously described [15]. Briefly, pregnant mice were sacrificed by schedule 1 method three days after first plug formation together with their virgin age-matched control littermates (12–13 weeks of age). Thoracic and inguinal mammary glands from one flank were dissected and collected into DMEM-F12 medium (Life Technologies, Paisley UK), while glands from the other flank were processed for paraffin-embedding. Glands were finely minced and digested with 2.5 mg/ml (w/v) collagenase type II (Sigma-Aldrich Ltd., St. Louis, USA) and Trypsin (0.2%) (Sigma) in DMEM-F12 medium for 30 min, using a shaking incubator set at 37 °C and 100 rpm. Free genomic DNA was removed using 2units/ml DNase I (Sigma), cells were centrifuged and resuspended thoroughly in serum-free DMEM-F12 medium. Epithelial cells were separated from other cells by a series of pulsed centrifugations (5–6 times) and fibroblast-containing supernatant was then incubated in a 100 mm tissue culture dish for 1 h at 37 °C and 5% CO_2_. Fibroblasts that stuck to the plate were washed 3 times and were either cultured or further purified for RNA extraction and amplification. For microarray analysis cells were gently trypsinised using 0.05% Trypsin/EDTA (Thermo Fisher Scientific, Waltham, MA, USA) and CD45^pos^ contaminants were removed using a CD45-Biotin antibody (Biolegend, San Diego, USA, Clone 30-F11) in conjunction with an EasySep Biotin Selection system (Stem Cell Technologies, Vancouver, Canada). The CD45^neg^ fibroblasts were collected by centrifugation and directly used for RNA extraction.

### Tissue culture

EpH4 cells and primary fibroblasts were grown at 37 °C in a humidified atmosphere with 5% CO_2_. Cells were grown in DMEM-F12/10% FBS (Thermo Fisher Scientific) media supplied with 2 mM L-glutamine (Thermo Fisher Scientific), 100 U/ml penicillin (Thermo Fisher Scientific) and 100 µg/ml streptomycin (Thermo Fisher Scientific).

### Epithelial-fibroblast co-culture

Growth factor-reduced Matrigel™ (BD Biosciences, Oxford, UK) was thawed on ice overnight. 50 μl were added to each well of a 96-well plate to cover the surface of the well and incubated at 37 °C for 30 min to solidify. 5000 cells per well (EpH4 cells and fibroblasts 1:1) were re-suspended in DMEM-F12/serum-free media mixed with 5% Matrigel and plated on top of the Matrigel layers. Cells were grown in triplicate in serum-free media overnight. Media was then replaced every other day with DMEM-F12/5% FBS for 7–8 days before microscopic analysis.

For structural analysis, spheroids were categorized according to their size and degree of branching to 5 distinct types: small unbranched, small branched, large unbranched, large branched and large highly branched (structures with secondary branching). For quantification, 10 × bright field objective was employed to count the structures directly from under the microscope in 4 representative fields per condition in triplicate experiments to avoid local differences within the 3D culture. Representative pictures were captured with 20 × objective for reference. Statistical analysis was performed on the mean percentage of the different structures between the conditions using the student T-test function in Excel.

### RNA isolation and amplification

RNA was extracted and amplified as described previously [15], using Direct-zol™ RNA MiniPrep (Zymo Research, Irvine, USA) for cultured cells (> 10,000 cells) or Direct-zol™ RNA MicroPrep (Zymo Research) for freshly isolated fibroblasts (500–2,000 cells) as per manufacturer’s instructions. RNA was eluted in RNase-free water and quantified using the NanodropND-1000 Spectrophotometer (Thermo Fisher Scientific). Quality was assessed using an Agilent bioanalyser (Agilent Technologies). An RNA 6000 Pico kit (Agilent, South Queensferry, UK) was used for quantification and assessment of RNA from small cell numbers.

RNA amplification was performed using Ovation® PicoSL WTA Systems V2 kit (NuGEN, San Carlos, USA) as per manufacturer’s instructions with slight modifications as described previously [15]. Briefly, equal volumes of RNA samples from individual pregnant and non-pregnant mice (minimum concentration 500 pg in 1 µl of maximum volume) in 5 µl of nuclease-free water were subjected to a cycle of 1st strand synthesis followed by a cycle of 2^nd^ strand synthesis. Double stranded product was separated from excess primers using the included magnetic bead-based system. For amplification, purified double stranded products were subjected to the SPIA amplification system (with RNase H and DNA polymerase) and the amplified ss-cDNA product was further purified using a PCR purification kit (QIAGEN Ltd., Manchester, UK) as per manufacturer’s instructions. Pure amplified product was reconstituted in nuclease-free water and quantified using a NanodropND-1000 Spectrophotometer (Thermo Fisher Scientific). Quality and distribution of the RNA and subsequent cDNA curves were assessed using an Agilent Bioanalyser (Agilent Technologies).

### Microarray hybridisation

After amplification, cDNA samples were labelled using an Encore Biotin Module (NuGEN) as per manufacturer’s instructions. 1.5 µg of cDNA was subjected to uracil-DNA glycosylase (UNG) treatment to remove uracil base incorporation from the amplification process, followed by one step of biotin incorporation. Samples were purified with a PCR purification kit (QIAGEN Ltd.) and samples were kept at − 20 °C until hybridisation.

cDNA samples were hybridised to MouseWG-6 v2.0 Expression BeadChip arrays (Illumina Inc., Little Chesterford, UK) as per manufacturer’s instructions. 1.5 μg of labelled cDNA of each sample in 10 μl was mixed with 20 μl of hybridisation buffer in nuclease-free tubes and pre-heated at 65 °C in a thermocycler for 5 min. Six pre-heated samples (three pregnancy-associated and three control virgin) were incubated in an incubation chamber with humidity control buffer at 48 °C for 16 h. The bead-chip was carefully de-sealed, washed and blocked in E1 blocking buffer for 10 min with rocking. The bead-chip was blocked in E1 buffer with a 1:1000 dilution of streptavidin-Cy3 (of 1 mg/ml) for 10 min, followed by a washing step for 5 min. Finally, the bead-chip was spun at 275 g for 4 min at 25 °C to dry and scanned with a microarray scanner (Illumina Inc.) via the decode file (.dmap) provided with the chip.

### Microarray analysis

Scanner data was transferred to Genome Studio software (Illumina Inc.) for hybridisation quality control and general data analysis. Quality control included sample-independent and sample-dependent assessments. Exported data were further analysed using R-software/ranking products (RP) module [18] (The R Foundation).

### qRT-PCR

cDNAs were produced with the RNA amplification kit or with Superscript II (Thermo Fisher Scientific). Each reaction mixture contained 1 μl (0.25 μM) probe, 1 μl primers mixture (7.2 μM of both forward and reverse primers), 10 μl of 2 × LightCycler® 480 TaqMan Master Mix (Roche, Basel, Switzerland), 5 μl diluted sample and nuclease-free water to a final reaction volume of 20 μl. Primers (Sigma): *Col3a1*: Fwd 5′-gtcctgctggaaaggatgg-3′, Rev 5′-ctggaggtccaggcagtc-3′ (Probe #80); *Col18a1*: Fwd 5′-gctcaccagtttggtcttgc-3′, Rev 5′-ccacctcctcagcaacattc-3′ (Probe #21); *Gpc1*: Fwd 5′-aggcagagatctcgggtga-3′, Rev 5′-gctctccagctccattcg-3′ (Probe #25); *Alx4*: Fwd 5′-gacacactaccctgatgtgtatgc-3′, Rev 5′-tccctctttcgccacttg-3′ (Probe #32); *Wisp2*: Fwd 5′-cccactgatccatcttctgg-3′, Rev 5′-tgtccaaggacaggcacag-3′ (Probe #6); *Tnc*: Fwd 5′-gggctatagaacaccgatgc-3′, Rev 5′-catttaagtttccaatttcaggttc-3′ (Probe #76); *Vcan:* Fwd 5′-cactggctgtggatggtg-3′, Rev 5′-cagcggcaaagttcagagt-3′ (Probe #62), *β-actin*: Fwd 5′-aaggccaaccgtgaaaagat-3′, Rev 5′-gtggtacgaccagaggcatac-3′ (Probe #56). PCR reaction was carried out using a LightCycler® 480 Instrument (Roche) under standard conditions. Relative RNA expression was normalised to the house-keeping gene actin and the data were presented as % of actin.

### Haematoxylin–eosin staining

5 μm FFPE-mouse mammary gland sections were immersed 3 × in xylene for 5 min, 3 × in absolute ethanol for 2 min, then rinsed in distilled water. After incubation in haematoxylin (Sigma) for 2 min, slides were washed under running tap water and dipped in Scott’s Tap water for 30 s. Slides were transferred to eosin (Dako, Eli, UK) for 2 min and rinsed thoroughly using running tap water. Stained tissue sections were de-hydrated through increasing concentrations of ethanol, immersed in fresh xylene for 3 min and finally mounted using Pertex mounting medium (Cell Path, Newtown, UK).

### Immunohistochemistry (IHC)

IHC was generally performed as described in [19]. 5 μm sections were routinely cut from formalin-fixed, paraffin wax-embedded mammary glands. Sections were deparaffinised for 10 min in two changes of xylene, hydrated in descending concentrations of ethanol and then immersed in water. Sections were incubated in 3% (v/v) hydrogen peroxide for 10 min to quench endogenous peroxidases; antigen retrieval was then performed using 1 mM EDTA buffer (pH 8) under high pressure. Sections were subjected to blocking step using pre-diluted 2.5% horse serum for 20 min, then incubated with primary antibody for 2 h at RT in a humidified chamber. All antibodies were diluted to their final concentrations using Antibody Diluent (Dako). Sections were then washed with PBS-0.1% Tween 20 (Sigma) three times for 10 min and incubated with polyclonal anti-rabbit (HRP labelled) secondary antibody (Vector Laboratories Ltd., Peterborough, UK) for 30 min. Primary antibodies used: anti-FBLN2 1:10,000 [20]; anti-VCAN 1:150 (Millipore/Chemicon AB1033, Burlington USA); anti-Col I (Abcam, Cambridge, UK, #ab21286); anti-Col IV (Abcam, #ab6586), anti-Col VI (Millipore/Sigma, #ab7821); anti-AGRN (R&D Systems, Abingdon, UK; #AF550); anti-FBLN5 (Abcam, #ab109428); anti-BMP1 (Abcam, #ab118520); anti-ALX4 1:150 (Sc-33643, Santa Cruz, Dallas, USA). Sections were washed with PBS-0.1% Tween 20 three times for 10 min and incubated in diluted (1 drop in 1 ml of Antibody Diluent) DAB + Chromogen (Vector Laboratories Ltd.) for 4 min. Stained tissue sections were counterstained with haematoxylin, dehydrated through ascending concentrations of ethanol and cleared with xylene before mounting with cover slips using Pertex mounting medium (Cell Path). Sections were imaged and scanned using a NanoZoomer Digital Pathology scanner (Hamamatsu Photonics UK Limited, Welwyn Garden City, UK) at 20 × magnification.

### Kaplan–Meier analysis

Kaplan–Meier analyses were performed using the Gene Expression-Based Outcome for Breast Cancer Online (GOBO) tool [17] from Lund University and KM-Plotter [21]. This tool uses RNA expression data from 1881 breast tumour samples generated on Affymetrix U133A microarrays in 11 independent studies that are freely available from the Gene Expression Omnibus (GEO; please refer to [17] for more detail). To assess prognostic power of individual RNAs (Kaplan–Meier analysis), the ‘Gene Set Analysis—Tumors’ function within GOBO was used. Distant metastasis-free survival (DMFS) and recurrence-free survival (RFS) were selected as end points, respectively, with a 10-year cut-off. The selected number of quantiles was set to ‘2’. LogRank *p*-values were adjusted for multiple comparisons across all genes and 20 predefined subgroups of the total cohort using the Benjamini–Hochberg method, implemented in the p.adjust function of R [22]. Genes which remained significant in any subgroup were included in the final 18-gene signature. To assess ability of any of the identified RNA signatures for breast cancer cohort stratification, the ‘Sample Prediction’ analysis function within the GOBO analysis tool was used. ‘Day 3 pregnancy vs control’ fold-change ratios were used as expression centroids within the signatures (averages were used where more than one probe per RNA was present). Kaplan–Meier analysis was performed using correlative centroid prediction (Pearson) with a cut-off of 0 (all patients with expression profiles positively correlated to the direction and magnitude of changes observed in early pregnancy were included in one group; negative correlations formed the other group). DMFS or RFS were selected as end points with a 10-year cut-off. LogRank *p*-values of < 0.05 were again regarded as significant. Multivariate analyses were performed in the presence of ER-status, LN-status, grade, age and tumour size. Multivariate analyses in GOBO are implemented using the survival library in R [17]).

For KM-Plotter analysis, patient cohorts were split using combined median expression levels of all probes against the 18-gene signature with negative weighting for those genes associated with good prognosis in GOBO. Again, a 10-year cut-off was used, with DMFS (breast cancer set) and progression-free survival (all other cancer sets) chosen as endpoint.

## Results

### Early pregnancy induces a loosening collagen sheath and BM protein expression

**Fig. 1:**
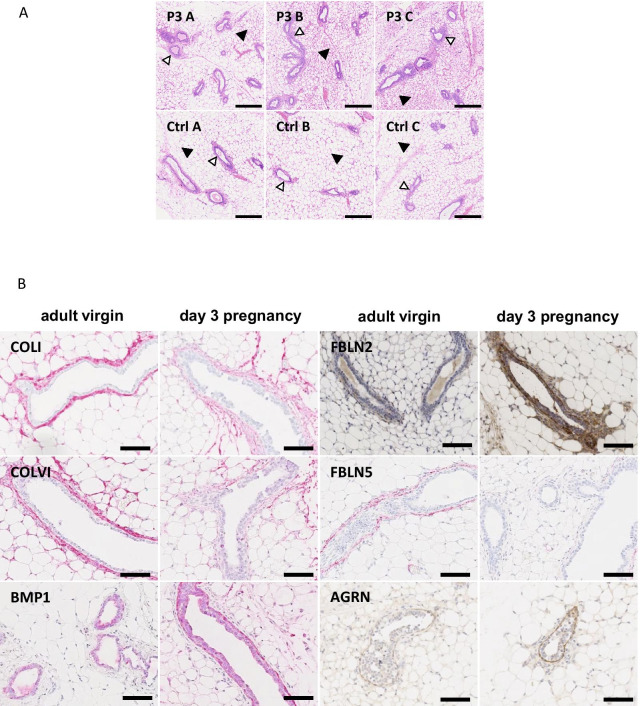
(**A**) Hematoxylin/eosin staining of representative (n = 3 animals) 3-days pregnant mouse inguinal mammary glands (**P3 A-C**) show the histological changes compared to age-matched adult virgin glands (**Ctrl A-C**) of denser stromal adipose tissue () and wider ECM layer around epithelium (). Scale bars are 250 µm (**B**) Immunohistochemical analysis of 3-days pregnant mouse (n = 1 animal, 4^th^ gland) mammary gland and age-matched adult virgin glands showing the changes of matrix- and basement membrane-associated proteins (COLI, COLVI, BMP1, FBLN2, FBLN5, and AGRN). Scale bars are 50 µm

The first microscopically observable histological changes occur two to three days after conception [13], including an overall denser stromal adipose tissue and more prominent ECM layer (Fig. 1A). To test whether these morphological changes were accompanied by an altered ECM protein expression, candidate proteins of the collagen sheath and BM were assessed by IHC in mammary glands of three days pregnant and age-matched virgin mice. Staining for fibrillary collagen (COL) I and COLVI was strong and highly localised around the ducts of non-pregnant adult mice. This staining appeared to become weaker and less defined in the glands of early pregnant mice (Fig. 1B). In contrast, bone morphogenetic protein (BMP) 1, involved in formation of new collagen fibrils, was predominantly detected around ductal epithelium of pregnant mice. This suggests a general loosening of the fibrillary collagen sheath at onset of pregnancy as new collagen bundles form. BM components like agrin (AGRN) and FBLN2 were noticably up-regulated in early-pregnancy mammary gland sections (Fig. 1B), consistent with our previous observation of upregulated FBLN2 and VCAN during early pregnancy [13]. Contrastingly, FBLN5 was more abundant around ducts from non-pregnant mice. Similar results were observed in the 3^rd^ gland of the same animals (Additional file 1: Figure S1). These results highlight widespread ECM remodelling and fibrillar collagen sheath loosening ahead of pregnancy-induced lateral ductal branch morphogenesis. Since fibroblasts play key roles in ECM remodelling, we next focussed our attention on the fibroblasts within the mammary gland of early pregnant mice.

### Pregnancy-associated fibroblasts induce branching of epithelial cells in vitro

**Fig. 2:**
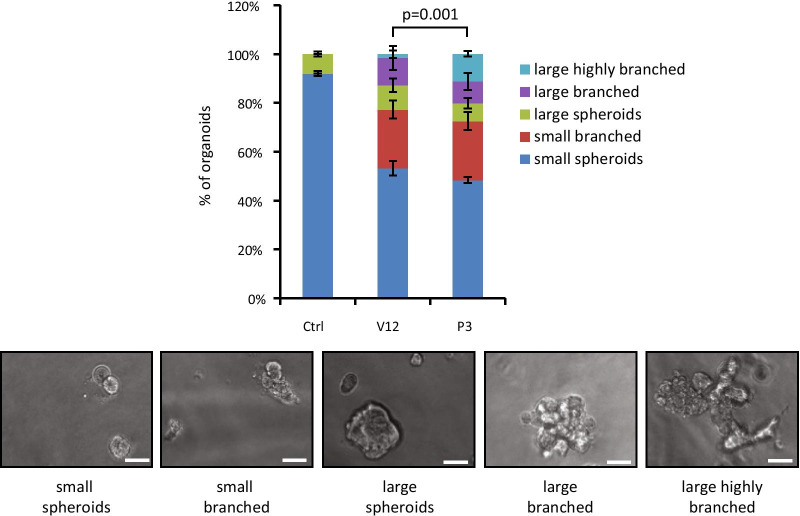
Percentage of specified epithelial 3D-growth patterns of EpH4 cells cultured within Matrigel (**Ctrl**) or co-cultured with mammary fibroblasts from 3-days pregnant (**P3**) or age-matched adult virgin mice (**V12**). Bars represent standard errors (n = 3 replicate experiments). Images below show representative images for each 3D structure classification of small spheroids (left), small branched (more elongated; 2^nd^ from left), large spheroids (at least 5-times larger than small spheroids; middle), large branched (branched but compact; 2^nd^ from right) to large highly branched structures (branched and extended; right). Scale bars are 50 µm. The *p*-value (0.001) shows the statistically significant difference in the number of large, highly branched structures between P3 and virgin control (Student T-test)

We hypothesised that if fibroblasts were involved in initiation of ductal branching in early pregnancy, isolated fibroblasts from early pregnant mice (pregnancy-associated fibroblasts (PAFs)) might initiate branching morphogenesis in vitro. We therefore isolated mammary fibroblast-enriched extracts from 3 days-pregnant and age matched non-pregnant control littermates. Both fibroblast-enriched extracts were initially cultured on plastic for 2–3 passages before co-culturing with mouse mammary epithelial EpH4 cells in Matrigel. While EpH4 cells alone formed mostly small spheroidal structures (Fig. 2), EpH4 cells grown in the presence of adult virgin mouse fibroblasts showed enlarged acini with limited branching. When EpH4 cells were grown in the presence of PAFs, significantly more enlarged, highly branched structures were detected (11% vs < 2%; *P* = 0.001; Additional file 2: Table S1). Hence, isolated PAFs and mammary fibroblasts from nulliparous mice showed significantly different branch initiation activities in vitro, which implied an altered gene expression pattern in these cells.

### Pregnancy induces a distinct RNA expression pattern in mouse mammary fibroblasts

We next aimed to identify the potential molecular mechanisms that could enable mouse mammary fibroblasts to induce branching morphogenesis in vivo by using whole genome transcriptome analysis. Freshly isolated, non-cultured primary fibroblast-enriched extracts were used to reflect the in vivo situation more closely. These were again isolated from inguinal and thoracic mammary glands dissected from one flank of 3 days-pregnant mice and from age-matched non-pregnant littermates. Pregnancy-associated morphological changes were confirmed by staining contralateral glands for FBLN2 as previously described [13], while increased COLIV staining confirmed further BM-associated changes (Additional file 3: Figure S2).

**Fig. 3:**
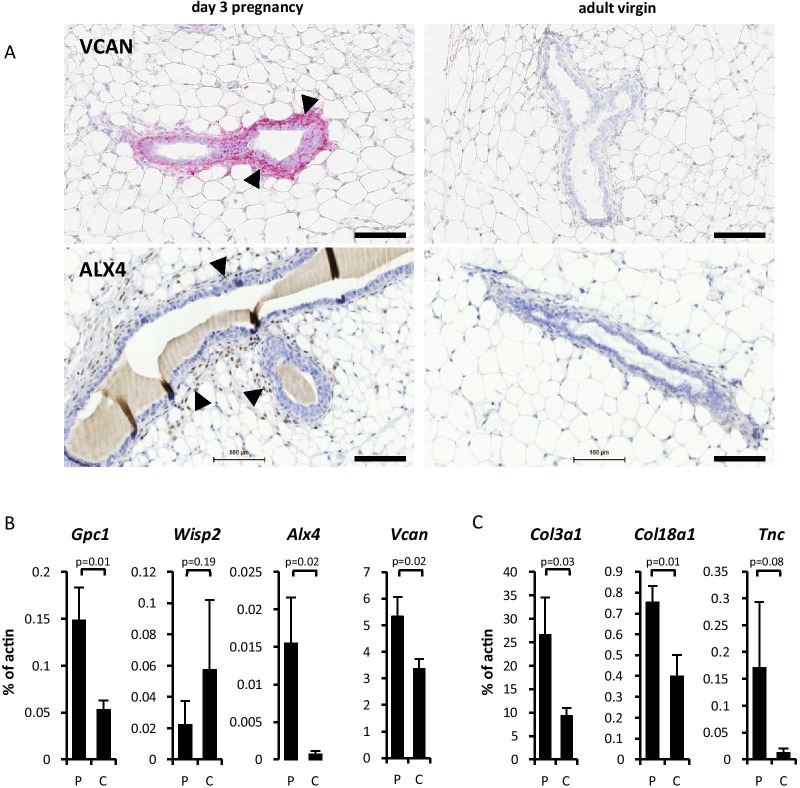
(**A**) Immunohistochemical staining for VCAN and ALX4 (arrow heads) around outgrowing ductal epithelium in 3-days pregnant mouse mammary glands and in glands from age-matched adult virgin glands (representative images, n = 4 (VCAN) and 3 (ALX4) animals respectively; see also Additional file 6: Figure S4). Scale bars are 100 µm. (**B**) Confirmatory qRT-PCR results for selected RNAs (*Gpc1, Wisp2*, *Alx4*, and *Vcan*) in 3-days pregnancy-associated (**P**) and control fibroblasts (**C**). Bars represent standard errors (n = 3 animals). (**C**) Confirmatory qRT-PCR results for selected matrisome protein encoding RNAs *Col3a1*, *Col18a1*, and *Tnc*. Bars again represent standard errors (n = 3 animals)

Analysis of markers for fibroblasts (*Platelet-derived growth factor-receptor 1* (*Pdgfra*), *Col1a1*, *Col1a2*, *serpin family H member 1* (*Serpinh1*)*/heat-shock protein 47* (*Hsp47*), *vimentin* (*Vim*)), *S100a4*), myo-fibroblast and myoepithelial cells (*smooth muscle actin* (*Acta2*)), myoepithelium (*keratin* (*Krt*) *5 and 14*), luminal epithelium (*Krt8 and 18*), vascular endothelium (p*latelet endothelial cell adhesion molecule* (*Pecam1*)/*Cd31, VE-cadherin* (*Cdh5*)), macrophages (EGF module-containing mucin-like hormone receptor (*Emr1*)), and leukocytes (*Cd45/Protein tyrosine phosphatase, receptor type, C* (*PtprC*)) confirmed strong enrichment of fibroblast-associated RNAs in our extracts from both pregnant and virgin mice, with low cross-contamination with other cell types tested (Additional file 4: Figure S3). Differentially expressed RNAs were then ranked according to their *p*-value. 897 probes showed a change with *p*-value < 0.05, representing 840 genes. Table 1 shows the 50 most significantly changed probes ranked by *p*-values and grouped into up- and down-regulated genes (for full list please see Additional file 5: Table S2). Two of the top four differentially expressed RNAs encoded proteins already known to be expressed in mammary stroma of pubertal and pregnant mice, which are necessary for mammary gland outgrowth and/or branching: *glucocorticoid receptor DNA-binding factor 1* (*Grlf1*) [23], and *aristaless-like 4* (*Alx4*) [24]. The list further included the proteoglycan *Vcan*, which we had previously described to be specifically detected in the stroma of pubertal outgrowing mammary epithelium and during early pregnancy [13]. IHC confirmed this specific expression, as well as expression of ALX4 protein in stromal cells surrounding ductal epithelia during early pregnancy (Fig. 3A; Additional file 6: Figure S4). Differential expression was further confirmed by qRT-PCR for a selection of identified RNAs (*Alx4, Gpc1, Vcan, Wisp2/Cnn5*), though *Wisp2/Ccn5* did not reach statistical significance (*P* = 0.19) (Fig. 3B).

**Table 1:** Top 50 differentially expressed RNAs (using RankP)

Probe_ID	Gene symbol	*p*-values	Fold change Preg/Ctrl	Median Preg	Median Ctrl
ILMN_2766542	*Grlf1*	0.0001	5.57	571.11	102.54
ILMN_1241214	*Myh4*	0.0001	5.05	293.53	58.09
ILMN_2487824	*Alx4*	0.0001	4.23	666.18	157.52
ILMN_2635784	*Gpc1*	0.0001	3.74	744.07	199.17
ILMN_2936517	*Bcl2l2*	0.0002	4.74	749.98	158.23
ILMN_1239430	*Mrc1*	0.0002	3.25	331.29	101.97
ILMN_2647028	*Copz2*	0.0002	2.84	729.06	256.57
ILMN_2930819	*Acd*	0.0003	3.20	1064.86	333.05
ILMN_1247540	*Vcan*	0.0003	3.10	435.82	140.46
ILMN_2945275	*Rbm15*	0.0005	2.84	427.28	150.19
ILMN_2636443	*Bcl2l2*	0.0006	3.49	857.62	245.92
ILMN_2709782	*Mtap2*	0.0008	3.98	205.62	51.69
ILMN_1258929	*2610110G12RIK*	0.0008	3.60	214.99	59.71
ILMN_1230753	*B230399E16RIK*	0.0008	3.32	575.35	173.35
ILMN_1249517	*Tmem168*	0.0010	3.53	225.91	64.01
ILMN_2683802	*Nudt9*	0.0010	2.69	203.48	75.67
ILMN_1237186	*Spint1*	0.0011	2.67	152.09	57.02
ILMN_1238360	*LOC100042405*	0.0011	2.41	941.65	390.74
ILMN_1246127	*Prss23*	0.0013	2.34	483.67	206.61
ILMN_2678002	*Notch2*	0.0015	2.66	227.93	85.71
ILMN_2463181	*Tnc*	0.0016	2.38	317.34	133.06
ILMN_2723024	*BC004044*	0.0016	2.28	168.75	74.1
ILMN_2878274	*Copz2*	0.0017	2.18	604.97	277.56
ILMN_2840818	*Cmpk*	0.0018	2.52	970.31	384.49
ILMN_1226553	*Mtap2*	0.0020	3.65	154.53	42.28
ILMN_1237216	*LOC100047827*	0.0020	2.14	831.45	388.45
ILMN_2697020	*Bat2d*	0.0021	2.12	503.2	237.3
ILMN_2670344	*Lrrn2*	0.0022	2.26	139.51	61.62
ILMN_2486906	*Wisp2*	0.0001	− 3.31	140.62	466.12
ILMN_2597606	*Gjc2*	0.0002	− 3.75	66.05	247.37
ILMN_2684370	*Igk-c*	0.0002	− 4.42	57.87	255.67
ILMN_1243031	*Trrp1*	0.0006	− 2.33	51.06	118.96
ILMN_1236368	*Orai1*	0.0007	− 2.11	79.89	168.37
ILMN_1232263	*Kcnk5*	0.0009	− 2.33	255.3	594.28
ILMN_1231553	*Bdh1*	0.0009	− 2.86	49.33	141.27
ILMN_2756435	*Cebpb*	0.0011	− 2.56	705.42	1802.36
ILMN_2906489	*Mocos*	0.0011	− 2.65	149.61	396.1
ILMN_2704562	*LOC100047628*	0.0011	− 4.67	58.56	273.36
ILMN_2968907	*Tipin*	0.0012	− 2.32	164.69	381.45
ILMN_3099265	*Anapc11*	0.0013	− 3.42	60.7	207.32
ILMN_2686327	*Gas6*	0.0014	− 1.63	704.55	1151.77
ILMN_1240289	*BC023744*	0.0014	− 2.07	138	286.23
ILMN_2760019	*Cxcl13*	0.0015	− 2.70	99.75	269.81
ILMN_2907214	*Tcea3*	0.0016	− 2.27	75.55	171.15
ILMN_2687794	*Tbx15*	0.0016	− 2.32	307.36	714.05
ILMN_1239386	*Galntl2*	0.0016	− 2.52	115.11	290.55
ILMN_1246999	*LOC677205*	0.0018	− 2.04	194.58	396.1
ILMN_1232824	*D430003P20RIK*	0.0018	− 2.82	199.47	562.53
ILMN_2601020	*Cnot10*	0.0021	− 2.32	144.9	335.67
ILMN_2890935	*Avpr1a*	0.0021	− 3.19	121.21	386.73

The table shows the top 50 hits (**Probe ID**) of RNAs that show significant (*p* < 0.05) differential expression (**Fold-change**) in fibroblast from 3-days pregnant (**Preg**) compared to virgin control (**Crtl**) mice, using median signal intensities from 3 individual experiments (biological replicates) and RankP software [18]. Probes have been ranked by decreasing fold-change and increasing *p*-value

### Pregnancy-associated gene expression changes identify an ECM-remodelling programme

To identify those factors most likely to affect the described morphological changes seen in the mammary gland of early pregnant mice, we next focussed on the RNAs encoding proteins of the matrisome as defined previously [16, 25]. Filtering the 897 probes identified 74 probes (8.24%), representing 64 differentially expressed core-matrisome and matrisome affiliated genes (Table 2). Again, qRT-PCR confirmed differential expression of selected RNAs (*Col18a1, Col3a1, Tnc*) within this table (Fig. 3C). This list described a complex programme of collagen remodelling, growth factor signalling and induced BM formation. STRING analysis showed a tight network with enrichment of factors associated with ECM organisation, collagen biosynthesis and cell motility, as well as cell adhesion, and glycosaminoglycan and heparin binding activities (Additional file 7: Figure S5, Additional file 8: Table S3).

**Table 2:** Matrisome-encoding RNAs with differential expression in PAFs

Probe ID	Gene symbol	*p*-values (< 0.05)	Fold change Preg/Ctrl	Probe ID	Gene Symbol	*p*-values (< 0.05)	Fold change Preg/Ctrl
**UP**				ILMN_1225835	*Mfap5*	0.0296	1.75
ILMN_2635784	*Gpc1*	0.0001	3.74	ILMN_2774315	*Col13a1*	0.0300	2.21
ILMN_1247540	*Vcan*	0.0003	3.1	ILMN_1256970	*Adamts18*	0.0323	1.71
ILMN_1246127	*Prss23*	0.0013	2.34	ILMN_2617087	*Egln1*	0.0347	1.84
ILMN_2463181	*Tnc*	0.0016	2.38	ILMN_1227502	*Il15ra*	0.0357	1.53
ILMN_2753809	*Mmp3*	0.0023	2.12	ILMN_1230599	*Adam23*	0.0362	1.78
ILMN_1243254	*Adam12*	0.0030	2.24	ILMN_3008858	*Ctsc*	0.0371	1.94
ILMN_2463180	*Tnc*	0.0036	2.4	ILMN_3158499	*Mdk*	0.0410	1.54
ILMN_2683958	*Col3a1*	0.0055	1.96	ILMN_2766028	*Postn*	0.0452	1.67
ILMN_2455596	*Adamts18*	0.0060	3.23	ILMN_2492264	*Wisp1*	0.0467	1.81
ILMN_2819929	*Plod2*	0.0064	1.57	ILMN_2688912	*F10*	0.0485	1.9
ILMN_1216882	*Postn*	0.0068	1.9	ILMN_2725927	*Serpina3g*	0.0488	1.88
ILMN_2769918	*Timp1*	0.0081	1.72	ILMN_2680415	*Sema6d*	0.0493	1.61
ILMN_1234487	*Angpt2*	0.0086	2.44	ILMN_2710698	*Fgf21*	0.0498	1.53
ILMN_1213850	*Col4a3*	0.0087	1.82	**DOWN**			
ILMN_3103896	*Timp1*	0.0091	1.78	ILMN_2486906	*Wisp2*	0.0001	− 3.31
ILMN_1221670	*Col5a2*	0.0092	2.29	ILMN_2686327	*Gas6*	0.0014	− 1.63
ILMN_2728729	*Sdc4*	0.0093	1.82	ILMN_2760019	*Cxcl13*	0.0015	− 2.7
ILMN_2735184	*Col18a1*	0.0116	2.71	ILMN_1231689	*Sfrp1*	0.0046	− 2.21
ILMN_2769884	*Igf1*	0.0119	1.43	ILMN_1229370	*Podn*	0.0094	− 2.85
ILMN_1253797	*Slit2*	0.0131	2.02	ILMN_1249485	*S100g*	0.0095	− 2.14
ILMN_2460257	*Adam10*	0.0154	1.7	ILMN_1219335	*Igfbp3*	0.0098	− 1.78
ILMN_2617090	*Egln1*	0.0166	1.84	ILMN_2851288	*Ngfr*	0.0121	− 1.73
ILMN_2731901	*S100a4*	0.0171	1.69	ILMN_1238597	*Omd*	0.0132	− 1.93
ILMN_2715840	*C1qc*	0.0172	1.79	ILMN_2595664	*Dhh*	0.0139	− 1.6
ILMN_1240458	*Mfap2*	0.0188	1.47	ILMN_2697220	*Figf/Vegfd*	0.0148	− 1.4
ILMN_2901626	*Tnfref21*	0.0212	1.95	ILMN_2773348	*Bmp8a*	0.0148	− 2.43
ILMN_2617086	*Egln1*	0.0224	1.69	ILMN_2461098	*Plxnb2*	0.0164	− 1.65
ILMN_2718204	*Clec11a*	0.0235	1.17	ILMN_2910653	*Bmp8a*	0.0183	− 2.35
ILMN_2671344	*Adamts12*	0.0242	1.65	ILMN_1251096	*Spock2*	0.0213	− 1.74
ILMN_1213777	*Fgf10*	0.0245	1.61	ILMN_2834379	*Tgfbi*	0.0252	− 1.73
ILMN_1260125	*Fn1*	0.0252	1.75	ILMN_1259632	*Col20a1*	0.0253	− 2.07
ILMN_2770429	*Plod2*	0.0252	1.73	ILMN_1252506	*Fbn1*	0.0261	− 2.63
ILMN_2638114	*Ptn*	0.0259	1.37	ILMN_2727503	*Igfbp3*	0.0297	− 2.63
ILMN_1239380	*Leprel2*	0.0280	1.71	ILMN_2507286	*Tnfsf13b*	0.0338	− 1.55
ILMN_2659151	*Thbs1*	0.0290	1.15	ILMN_2718401	*Htra3*	0.0392	− 1.82
ILMN_2486186	*Tnfrsf1b*	0.0291	1.75	ILMN_1234111	*Vtn*	0.0392	− 1.48
ILMN_1234413	*Adamts12*	0.0293	1.5	ILMN_1214439	*Ltbp1*	0.0426	− 1.51

The table shows the 74 probes (**Probe ID**), representing 64 different RNAs, that encode proteins associated with the Matrisome [16] and are differentially expressed (*p* < 0.05) in fibroblasts from 3-days pregnant mouse mammary glands (**Preg**) compared to fibroblasts isolated from age-matched virgin control glands (**Ctrl**). Probes have been ranked by decreasing fold-change and increasing *p*-value

**Fig. 4:**
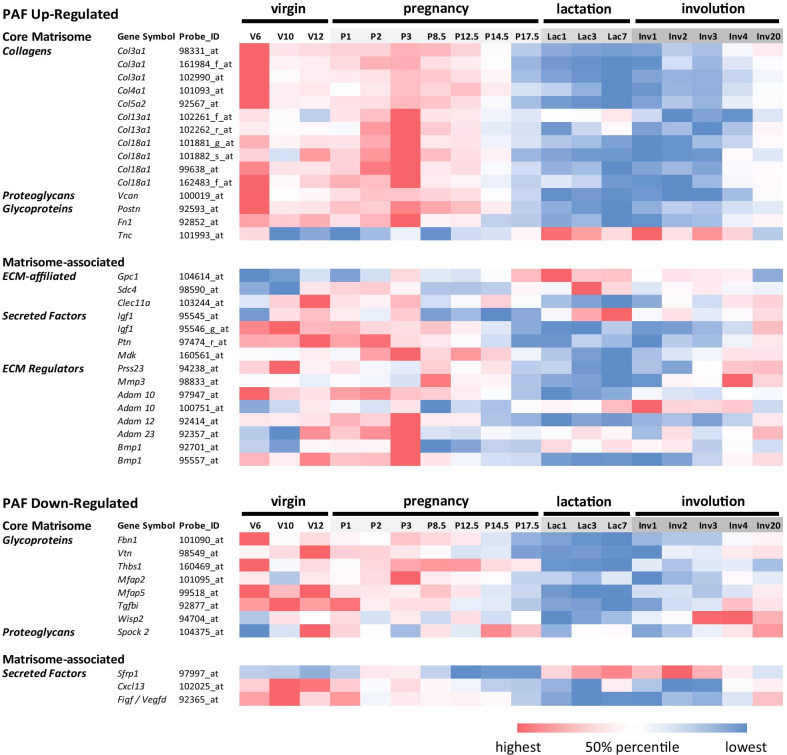
Microarray signal intensities of identified PAF-associated core matrisome and matrisome-associated RNAs in whole Balb/C mouse mammary glands at different mammary gland developmental stages (Virgin (**V**) at 6, 10 and 12 weeks, Pregnancy (**P**) days 1, 2, 3, 8.5, 12.5, 14.5 and 17.5, Lactation (**Lac**) days 1, 3 and 7, and Involution (**Inv**) days 1, 2, 3, 4 and 20) identified in a previous dataset [26]. Colour intensities reflect signal intensities relative to the median (50% percentile) of each RNA across all developmental time points (red: above; blue: below)

To establish if expression of identified matrisome RNAs is specific to early pregnancy, we assessed expression levels at other stages of mammary gland development (puberty, adult virgin, early-, mid-, and late pregnancy, lactation, and involution) using previously obtained microarray data from whole BALB/c mouse mammary glands [26]. Data were available for 33 of the 64 Matrisome RNAs (Fig. 4). 18 of 24 RNAs upregulated in PAFs showed the strongest abundance during times of epithelial outgrowth, puberty (V6) and/or early pregnancy (P3) (*Adam10, Adam12, Adam23, Bmp1, Col3a1, Col5a2, Col13a1, Col18a1, Gpc1, Vcan, Col4a1, Agrn, Postn, Fn1, Mfap2, Mfap5, Tnc, Mdk*). Most were down regulated at mid-pregnancy at onset of alveolar differentiation. RNA abundances therefore coincided mostly with ductal outgrowth and branching initiation. Similarly, 5 of 9 genes (*Vtn, Cxcl13, Figf/Vegfd, Spock2* and *Tgfbi*) whose RNAs were down regulated in PAFs, were also reduced in P3 glands compared to non-pregnant adult glands.

### The matrisome signature of PAFs predicts distant-metastasis free survival (DMFS)

**Fig. 5:**
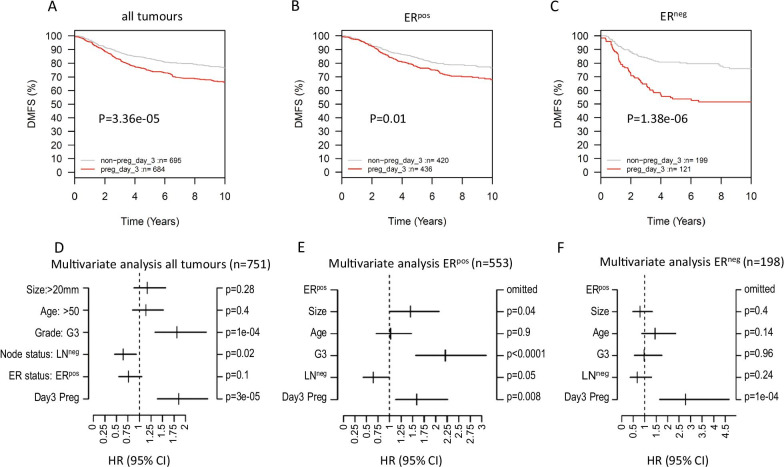
Kaplan–Meier analyses generated using the GOBO online tool and centroid correlation clustering for the 64-gene matrisome signature (endpoint: distant metastasis free-survival (DMFS)) in (**A**) all breast cancers, (**B**) or the ER-positive (**ER**^**pos**^) and (**C**) ER-negative (**ER**^**neg**^) subgroup. (**D**–**F**) Multivariate analyses for the 64-gene signature (**Day3 Preg**) within same groups as above in the presence of other clinical parameters: size > 20 mm (**Size**), age > 50 years (**Age**), grade (**G3**), LN-status (**LN**^**neg**^) and ER-status (**ER**^**pos**^)

For normal and cancerous epithelium to grow into the surrounding stroma, both require stromal remodelling. Tissues often use the same or similar molecular mechanisms to drive similar morphological changes. We therefore hypothesised that the molecular mechanisms associated with tissue remodelling during early mammary lateral branching may also operate during human breast cancer progression, enabling and/or supporting cellular invasion and further metastatic spread. If this was the case, we would expect the RNA expression patterns found in our PAFs to be found at least in part in breast cancers with a higher risk of progression and metastasis formation. To test this hypothesis, we assessed whether expression of our 64 gene matrisome RNA signature correlated with metastatic spread in 1881 breast cancer patients, using the Gene expression-based Outcome for Breast cancer Online (GOBO) webtool [17]. Distant metastasis-free survival (DMFS) was used as endpoint with a 10-year cut-off point. Fold-change values (preg vs ctrl) in expression of each gene from our array analysis were used as expression centroids (Table 2). Kaplan–Meier analysis showed that the 64 gene PAF matrisome signature was a strong univariate prognostic indicator of DMFS (LogRank *P* = 3.36e−5) for all breast cancers. The signature remained significant in multivariate analysis including age, tumour size, grade, ER-, and LN status (HR = 1.85, 95% CI: 1.39–2.48, *P* = 2.74e−5) (Fig. 5, Additional file 9: Figure S6). Similar results were obtained when recurrence-free survival was used as an endpoint (Additional file 10: Figure S7). Univariate subgroup analysis showed that this signature predicted DMFS in the basal (LogRank *P* = 0.047) and HER2-positive cancer cohorts (LogRank *P* = 0.004), though not luminal A or B, or normal-like cancer subgroups (Additional file 9: Figure S6). Correspondingly in multivariate analyses, the signature was more powerful in the ER^neg^ cohort (HR = 2.78 (1.65–4.68), *P* = 1e-04) than in the ER^pos^ cohort (HR = 1.59 (1.13–2.26), *P* = 0.008) (Fig. 5). Additionally, the signature showed prognostic power in all histological grades (grade 1: LogRank *P* = 0.008; grade 2: LogRank *P* = 0.008; grade 3: LogRank *P* = 0.005) (Additional file 9: Figure S6). Therefore, the signature is a prognostic indicator independent of grade.

### An 18-RNA matrisome signature shows significantly increased prognostic power

To identify the most significant contributors to the signature’s prognostic power, each of the 64 genes was tested individually within the GOBO breast cancer dataset. 52 of them were recognised in this dataset. 48 of 52 reached significance (*p* < 0.05) to stratify patient groups in at least one defined breast cancer subgroup. Forty-two were consistently associated with either higher or lower levels of DMFS within the subgroups. After multiple testing correction across all gene- and subgroup-analyses, 18 RNAs retained an adjusted *p*-value of < 0.05 for at least one breast cancer subgroup (Additional file 11: Figure S8A). 11 of those 18 RNAs (*WISP2, CXCL13, POSTN, COL5A2, COL13A1, COL18A1, OMD, CLEC11A, FBN1, SFRP1, SPOCK2*) were either up-regulated in PAFs with high breast cancer expression of the human orthologues associated with poor prognosis, or down-regulated in PAFs with low breast cancer expression associated with poor prognosis, and therefore potential drivers of progression within the signature. Contrastingly, the other 7 RNAs (*VCAN*, *TIMP1*, *IGF1*, *SLIT2*, *TGFBI*, *CTSC*, *VTN*) had up-regulated expression of the RNAs in PAFs but high expression of the human orthologues in breast cancers was associated with better prognosis, or down-regulated in PAFs and low expression in breast cancers was associated with better prognosis (Additional files 11: Figures S8, Additional files 12: Figure S9). These differences might reflect the controlled nature of mammary epithelial branching morphogenesis versus the uncontrolled situation in metastasis.

Using again the fold-change expression values of these 11 + 7 RNAs in PAFs, the univariate prognostic power of this combined gene set was comparable to the initial 64 gene set (LogRank *P* = 2.45e−06). However, a signature using only the above-mentioned 11 RNAs showed a strongly increased prognostic power (Log-rank *P* = 7.66e−12). Similarly, a signature using only the residual seven RNAs also showed stratification ability, but in the opposite direction (LogRank *P* = 6.42e−09) (Additional file 11: Figure S8B, C).

**Fig. 6:**
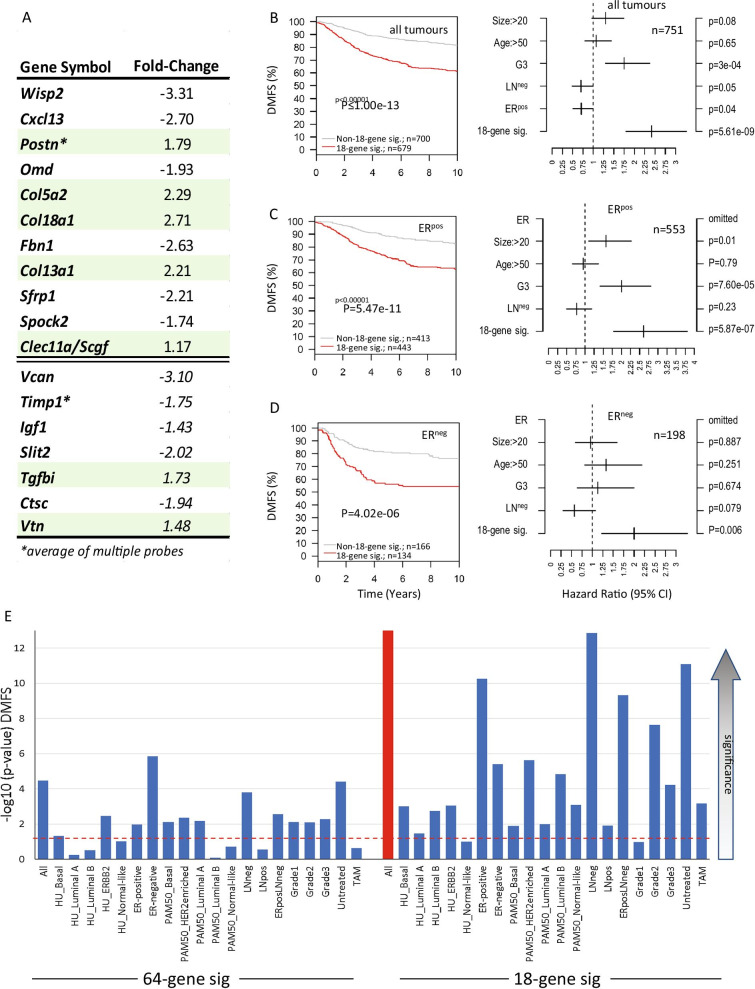
(**A**) List of the 18 genes of the shortened matrisome-signature after multiple testing correction with fold-change expression values used for the 18-gene signature. The average fold-change of multiple probes was used for Postn and Timp1 (*). The expression values for the seven RNAs below the double lines have been reversed to reflect their tumour suppressor-like behaviour as described in the text. (**B**) Kaplan–Meier analyses (DMFS) generated using the GOBO online tool and centroid correlation clustering for the shortened 18-gene matrisome signature (18-gene sig.) in all breast cancers, (**C**) the ER-positive (ER^pos^) and (**D**) ER-negative (ER^neg^) subgroup with associated multivariate analyses in the presence of other clinical parameters: size (> 20 mm), age (> 50 years), high grade (G3), LN-status (LN^neg^) and ER-status (ER^pos^). (**E**) Bar graph comparing LogRank *p*-values (-log10) from each KM-analysis in the total breast cancer set and all available subgroups obtained for either the 64-gene or 18-gene signature (DMFS). The actual *p*-value for the 18-gene signature for all tumours (red bar) was rounded down by the software to *p* = 0 as *p* ≤ 1e−13. Red dotted line shows LogRank *p* = 0.05 with increasing significance above the line

To improve the prognostic power of the 18 gene signature, expected centroids for the other seven RNAs were reversed. As a result, the prognostic power was significantly strengthened for all tumours (LogRank *P* $\le \hspace{0.17em}$1e−13) (Fig. 6A, B) and remained significant in multivariate analysis (HR = 2.42 (1.8–3.26), *P* = 5.61e−09) (Fig. 6B; Additional file 13: Figure S10). Furthermore, this signature was now able to identify tumours with poor DMFS in nearly all subgroups analysed (ER^pos^, ER^neg^, LN^pos^, LN^neg^, ER^pos^/LN^neg^, Grade-2, -3, Luminal A, Luminal B, ErbB2, Basal, untreated and tamoxifen-treated) (Fig. 6C-E; Additional file 14: Figure S11) with the exception of Normal-like cancers (*P* = 0.10) and Grade-1 cancers (*P* = 0.10) (Fig. 6E; Additional file 14: Figure S11). Similar, if slightly weaker, results were obtained when recurrence-free survival was used as end point (LogRank *P* = 3.08e-08; HR = 1.98 (1.55–2.52), *P* = 3.08e−08) (Additional files 13: Figure S10, Additional files 15: Figure S12).

### Verification of the signature’s prognostic power using the KM-Plotter database

**Fig. 7:**
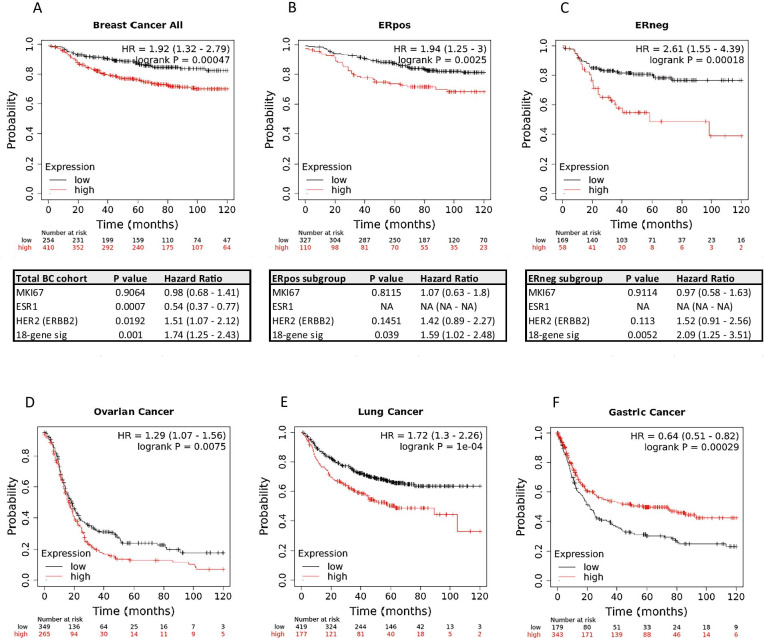
Kaplan–Meier analyses generated with KM plotter for the 18-gene signature in (**A**) all breast cancer patients, (**B**) ER-positive (**ERpos**) and (**C**) ER-negative cases (**ERneg**) using DMFS as endpoint and a 10-year cut-off. Patient cohorts were split using the combined median expression levels of all 18 genes with negative weighting for genes associated with good prognosis in the GOBO analysis. ‘High’ and ‘Low’ expression cut-offs were chosen automatically by KM-Plotter for best fit. Tables below each graph show the multivariate analysis results, performed in the presence of ER status (*ESR1*), Ki-67 (*MKi67*)- and HER2 status, and showing hazard ratios and 95%-confidence intervals. (**D**) Kaplan–Meier plots for the 18-gene signature, using progression-free survival as endpoint for the ovarian cancer set, and (**E**) time to first progression for the lung cancer dataset, as well as (**F**) for gastric cancer patients within KM-Plotter

To verify the prognostic power of our signature, it was further tested using ‘Kaplan–Meier Plotter’ [21]. In univariate analysis, this signature stratified breast cancer patients according to DMFS in all patients (HR = 1.92 (1.32–2.79), LogRank *P* = 0.00047) as well as in ER^pos^ (HR = 1.94 (1.25–3), LogRank *P* = 0.0025) and ER^neg^ subgroups (HR = 2.61 (1.55–4.39), LogRank *P* = 0.00018) (Fig. 7A-C). Multivariate analyses including ER status, Ki-67- and HER2 status confirmed that the signature remained a significant predictor of DMFS in all three tested cohorts (Fig. 7A-C).

To test for breast cancer-specificity of the signature, we analysed its prognostic power also in the gastric, ovarian and lung cancer datasets available in KM-Plotter. The signature had significant prognostic power in all datasets (ovarian cancer (HR = 1.29 (1.51–1.56), LogRank *P* = 0.0075); lung cancer (HR = 1.72 (1.3–2.26), LogRank *P* = 1e−04)). However, in gastric cancer, higher signature levels were associated with better prognosis in this cohort (HR = 0.64 (0.51–0.82), LogRank *P* = 0.00029) (Fig. 7D–F). Nevertheless, our data show that the described changes are not breast cancer-specific.

## Discussion

The mammary stroma has been recognised as an important determinant of epithelial growth and differentiation during normal development as well as cancer progression [1, 27]. Lateral branch formation and cancer invasion both require major remodelling of the surrounding BM and collagen sheath, creating an epithelial growth promoting environment. In both cases, fibroblasts play significant regulatory roles [11, 28]. We therefore hypothesised that by studying the gene expression changes in primary mammary fibroblasts during the initiation of pregnancy-associated lateral branching morphogenesis, we may identify stromal factors that control normal ductal branching and also regulate breast cancer invasion, and hence progression to metastatic disease. We have identified an 18-gene PAF-associated RNA signature that can now be used as a starting point for further biological tests, studying the functions of the associated proteins during mammary branching morphogenesis and breast cancer progression. It is so far unclear whether the identified RNAs reflect different levels of expression within cancer-associated fibroblasts or other stromal cells within the individual tumours, or within the cancer epithelium itself, e.g. through epithelial-mesenchymal transition-type changes. This can now be addressed by IHC and in-situ hybridisation in future studies. Importantly, our data show that the changes of RNA expression of these genes are associated with cancer progression in a significant number of breast cancers, and could therefore play important roles in the control of invasion and/or metastasis formation.

11 of the 18 genes showed similar expression patterns during early pregnancy (5 up- and 6 down-regulated) and in poor prognosis breast cancer, and therefore identified potential stromal regulators of tissue remodelling during normal development and breast cancer progression that occur in both biological settings. However, expression of the other seven RNAs in the PAFs behaved the opposite way to what was expected, being upregulated (*Timp1, Slit2, Igf1, Vcan, Ctsc*) or downregulated (*Tgfbi, Vtn*) in PAFs during pregnancy, but in the human breast cancers this expression pattern was associated with improved rather than poor DMFS (see Additional file 11: Figure S8C)*.* These RNAs therefore behaved diametrically opposed to our initial hypothesis. We hypothesise that these seven identified factors reflect some of the differences between the ‘controlled invasion’ of normal mammary branching morphogenesis and cancer cell invasion, and therefore may identify mechanisms that prevent mammary epithelial cells from growing uncontrollably into surrounding stroma. One unexpected finding was the correlation between increased *Vcan* RNA expression and better DMFS in ER^pos^ and LN^neg^ cancers as VCAN protein expression in peritumoral stroma of the breast has previously been associated with poor prognosis in LN^neg^ breast cancers [29]. That study analysed a mix of 60 grade 1–3 breast cancers in total with six showing increased VCAN staining. Unfortunately, no further clinical data (e.g. ER-status) or association of VCAN staining with grade were available for this patient group. This contradiction may reflect a divergence between RNA and protein expression. However, in the mouse mammary gland, VCAN is strongly co-expressed and colocalises with FBLN2 [13], a protein required for BM integrity, around newly outgrowing ducts during puberty and early pregnancy [14]. We have recently shown that in breast cancer FBLN2 together with COLIV is reduced in areas of invasion compared to neighbouring morphological normal tissue, with high *Fbln2* RNA levels showing significant association with better DMFS in breast cancers of low and intermediate grade in KM-Plotter. In contrast, in high grade cancers *FBLN2* RNA expression was associated with poor prognosis [14]. This could reflect different protein requirements at the various progression stages, where FBLN2 presence may suppress tumour invasion in the early stages but may enable cancer cells to survive and form metastases once invasion has occurred. This could either be through expression of those stromal proteins by the malignant cells themselves or by inducing their local microenvironment to express these proteins, as the tumour ECM is a product of both the tumour epithelial and stromal cells [30]. Hence, our results may reflect a similar association for VCAN.

Collagens form a key part of the extracellular matrix, requiring extensive remodelling during tissue turnover during cancer progression and development. This is reflected in our 18-gene signature, with three of five RNAs upregulated in PAFs and for which higher expression in breast cancers was associated with poor prognosis encoded collagen proteins (*Col5a2, Col13a1, Col18a1*). COLV is an essential regulator of collagen fibrillogenesis [31] and is expressed in breast cancer desmoplastic stroma in response to invasive carcinoma [32]. Consistent with our data, *COL5A2* expression itself is upregulated in epithelial cells of breast invasive ductal carcinoma compared to DCIS [33]. Similarly, COLXIII has been detected in several cancers at the invasive front [34] and its expression in breast cancers is associated with increased invasion and metastasis [35]. Interestingly, recent evidence has linked COL18A1 to the mammary stem cell-niche with *Col18a1*^−/−^ mice developing fewer terminal end buds and branch points. Oestrogen and progesterone induce WNT4, which activates the protease ADAM-TS18 in myoepithelial cells, leading to remodelling of the BM and activation of mammary stem cells through binding of ADAM-TS18 to COL18A1 in the stem cell niche [36]. It is therefore interesting to note that *Adamts-18* was also significantly induced in PAFs together with *Col18a1* (Table 2).

The Wnt-signalling pathway is an important activator of mouse mammary branching morphogenesis [37], and two further RNAs in our signature indicated an involvement of our PAFs in the activation of the Wnt-pathway and mammary stem cells: *Postn* and *Sfrp1*. *Postn* is necessary for correct collagen fibril assembly [38] and for metastatic colonisation, recruiting Wnt-ligands for cancer stem cell maintenance [39]. It has been detected in cancer-associated fibroblasts of invasive breast carcinoma [40, 41], and overexpression in human mammary epithelial cells enhances breast tumour growth and metastasis [38]. *Sfrp1* is a negative regulator of Wnt-signalling, which was downregulated in our PAFs, and reduced *SFRP1* was associated with poor DMFS. This is consistent with *SFRP1* being epigenetically silenced in ~ 75% of invasive breast cancers [42].

By focussing our study purely on the matrisome, our signature did not show any similarities with previously described prognostic RNA signatures, such as the Core Serum Response signature by Chang et al. [43], which was derived from cultured serum-activated fibroblasts. Our study deliberately avoided in vitro culturing, and instead used RNA from freshly isolated primary PAFs. Our RNA data therefore should reflect the in vivo situation more closely [44]. Further, in contrast to other published signatures [45], our signature is also not driven by descriptors of cellular proliferation and ER-signalling and is hence independent of grade.

In recent years, several molecular diagnostic RNA signatures for breast cancer progression have been developed and are now widely commercially available (for example Endopredict [46] and OncotypeDX [47, 48]). All of these have been specifically designed and approved to assess the risk of metastasis formation in early low-grade ER^pos^/HER2^neg^/LN^neg^ breast cancer patients, which represent 60–70% of all newly diagnosed cases. Since our signature performs particularly well in high grade, ER^neg^, and HER2^pos^ breast cancer cohorts, it might complement these established tools in providing crucial information about the risk of distant metastasis formation for therapy decision-making in these difficult to treat patient groups. Notably, the 18-gene set had prognostic significance using the different analysis methods of GOBO and KM plotter, and performed well when compared to Endopredict and OncotypeDX, using the GOBO Gene Set Analysis Tool (Additional file 16: Table S4), which allows for analysis of weighted expression (rather than the centroidal analysis method of ‘Sample Prediction’, as shown in Figs. 5 and 6). We acknowledge that the current comparison is imperfect. Nevertheless, our results show that our matrisome derived gene set performs better, particularly in HER2 and ER^neg^ and high-grade tumours than proliferation-associated signatures. Therefore, several approaches could now be taken to develop an optimised score.

## Conclusions

In summary, we identified potential new candidates involved in a complex system of stromal-controlled epithelial branching, and provide a testable novel dataset for further analyses of stromal-epithelial interaction and stromal-controlled breast cancer progression. In addition, we have provided a potential new tool to identify breast cancer patients, particularly within the ER^neg^ and HER2^pos^ cohorts that have a significantly altered risk of developing metastases, and may therefore be further developed into a diagnostic tool to aid therapy decision-making.

## Supplementary Information

**Additional file 1: Figure S1.** Immunohistochemical analysis of 3-days pregnant mouse (n = 1 animal, 3rd gland) mammary gland and age-matched adult virgin glands showing the changes of matrix- and basement membrane-associated proteins (COLI, COLVI, BMP1, FBLN5, and AGRN). Scale bars are 50 µm.

**Additional file 2: Table S1.** Table with total and average counts (and standard deviation) of identified 3D-structures from triplicate co-culture experiments. Student T-tests were performed to compare co-cultures with fibroblasts from **P3** and age-matched control (**V12**) mice.

**Additional file 3: Figure S2.** Immunohistochemical analysis using the contralateral inguinal mammary glands for validation of the specific expression of **FBLN2** around the outgrowing ductal epithelium as a marker of early pregnancy in all three mice used for the isolation of 3-days pregnancy-associated fibroblasts (**P3 A-C**) and their age-matched control counterparts (**Ctrl A-C**). Similar results were obtained for **COLIV**. Scale bars are 100 µm.

**Additional file 4: Figure S3.** Microarray signal intensities of markers for fibroblasts (*Pdgfra* (probe_ID ILMN_1235932), *Col1a1* (ILMN_2687872), *Col1a2* (ILMN_1253806), *Serpinh1* (*Hsp47;* ILMN_2822850), *Vim* (ILMN_2451022), *S100a4* (ILMN_2731901)), fibroblast/myoepithelial cells (*Acta2* (*smooth-muscle actin*; ILMN_2693895; ILMN_27103549)), myopepithelial cells (*Krt5* (ILMN_2740939), *Krt14* (ILMN_2722616)), luminal epithelial cells (*Krt8* (ILMN_1221157), *Krt18* (ILMN_2711267)), vascular/endothelial cells (*Pecam1* (*Cd31*; ILMN_2700982; ILMN_3147074), *Cdh5* (*VE-cadherin*)), macrophages (*Emr1* (ILMN_1216880; ILMN_2847787)), as well as leukocytes (*PtprC (Cd45*; ILMN_1212836; ILMN_2671984)) from PAFs (**Day3 Pregnancy**) and from fibroblasts isolated from age-matched virgin counterparts (**Adult Virgin**). Bars represent standard errors (n = 3 animals). Identically labelled columns represent the signal intensities from individual probes for the same RNA.

**Additional file 5: Table S2.** All 897 probes with differential abundance in pregnancy-associated fibroblasts (*p*-value < 0.05) as identified by RankP software analysis [18]. Probes have been ranked by decreasing fold-change.

**Additional file 6: Figure S4.** Immunohistochemical analysis using the contralateral inguinal mammary glands for validation of the specific expression of **VCAN** around the outgrowing ductal epithelium in the three mice used for the isolation of 3-days pregnancy-associated fibroblasts (**P3 A-C**) and their age-matched control counterparts (**Ctrl A-C**). Similar results were obtained for **ALX4**. Note that for ALX4 Ctrl A and P3 A show the same sections as in Fig. 3A. Scale bars are 100 µm.

**Additional file 7: Figure S5.** STRING analysis using the 64-gene matrisome genes showing their predicted protein–protein interactions. The table below shows the two most significantly over-represented Gene Ontology (GO) biological processes, molecular functions, and local network clusters with enrichment-level (**strength**) and false-discovery rate (**FDR**) for each grouping. The ‘Count in gene set’ numbers represent the number of genes within our signature associated with a particular GO group out of the total of genes associated with this group.

**Additional file 8: Table S3.** Table of enriched Biological Processes, Molecular Functions and Network Clusters in the 64 gene set as defined by STRING analysis.

**Additional file 9: Figure S6.** Kaplan–Meier analyses generated using the GOBO online tool for the 64-gene matrisome signature (DMFS) in all patients and each breast cancer subgroup classified based on ER status, Tumor grade, LN status and molecular subtyping according to Hu et al. [49–51]or PAM50 (Luminal A, Luminal B, Her-2, Basal and Normal-like) [52], as well as Tamoxifen treatment.

**Additional file 10: Figure S7.** Kaplan–Meier analyses generated using the GOBO online tool for the 64-gene matrisome signature (RFS) in all patients and each breast cancer subgroup classified based on ER status, Tumor grade, LN status and molecular subtyping according to Hu et al. [49–51]or PAM50 (Luminal A, Luminal B, Her-2, Basal and Normal-like) [52], as well as Tamoxifen treatment.

**Additional file 11: Figure S8.** (**A**) List of the multiple-correction adjusted *p*-values for those genes with a *p*-value of *p* < 0.05 in at least one breast cancer subtype. Colours show the association of either high (**red**) or low (**blue**) expression for each gene that is associated with poor DMFS. (**B**) List of the same RNAs with fold-change expression values between PAFs (**Preg**) and control (**Ctrl**) fibroblasts. The average fold-change of multiple probes was used for *Postn* and *Timp1* (*). (**C**) Kaplan–Meier analysis for gene signatures of either those 11 genes for which the expression (up or down) during pregnancy is also associated with poor DMFS in breast cancer (**11_gene_sig**; *WISP2, CXCL13, POSTN, COL5A2, COL13A1, COL18A1, OMD, CLEC11A, FBN1, SFRP1, SPOCK2*), and the seven genes for which the expression (up or down) during pregnancy is associated with improved DMFS (**7_gene_sig**; *VCAN*, *TIMP1*, *IGF1*, *SLIT2*, *TGFBI*, *CTSC*, *VTN*), or a combination of the two (**11 + 7_gene_sig**).

**Additional file 12: Figure S9.** Kaplan–Meier analyses generated using the GOBO online tool for each single gene of the shortened 18-gene signature (DMFS) in the total breast cancer dataset (showing non-adjusted *p*-values). Cut-offs of median expression for each gene were automatically chosen by GOBO for best separation into two survival groups.

**Additional file 13: Figure S10.** Multivariate Analysis for 18-gene signature (**18_gene_sig**) in the total breast cancer cohort (**Total BC cohort**), ER^pos^ (**ER_pos**) or ER^neg^ (**ER_neg**) cancer patients in the presence of other clinical parameters: size > 20 mm (**Size**), age > 50 years (**Age**), grade (**G3**), LN-status (**LN**^**neg**^) and ER-status (**ER**^**pos**^), using either DMFS or RFS as endpoint (10-year cut-off).

**Additional file 14: Figure S11.** Kaplan–Meier analyses generated using the GOBO online tool for the shortened 18-gene matrisome signature (DMFS) in all patients and each breast cancer subgroup classified based on ER status, Tumor grade, LN status and molecular subtyping according to Hu et al. [49–51]or PAM50 (Luminal A, Luminal B, Her-2, Basal and Normal-like) [52], as well as Tamoxifen treatment.

**Additional file 15: Figure S12.** Kaplan–Meier analyses generated using the GOBO online tool for the shortened 18-gene matrisome signature (RFS) in all patients and each breast cancer subgroup classified based on ER status, Tumor grade, LN status and molecular subtyping according to Hu et al. [49–51] or PAM50 (Luminal A, Luminal B, Her-2, Basal and Normal-like) [52], as well as Tamoxifen treatment.

**Additional file 16: Table S4.** Comparison of the 18-gene signature (**18_gene_sig**) with the 21 gene signature from **OncotypeDX** and the 8 gene signature from **Endopredict**, using ‘Gene Set Analysis’ tool in GOBO, which allowed for analysis of weighted expression as previously defined [46, 48]. DMFS was chosen as endpoint (10-year cut-off). *p*-values for Kaplan–Meier analysis are shown for the total cohort and each breast cancer subgroup. Yellow highlights subgroups for which only the 18-gene signature shows a significant stratification (*p* < 0.05), while green highlights those subgroups, in which the 18-gene signature showed significance with the lowest *p*-value.

## Data Availability

The datasets generated and analysed in the current study are available in the GEO repository: GSE167438.

## Abbreviations

AGRN: Agrin
ALX4: Aristaless-4
BM: Basement membrane
CAF: Cancer-associated fibroblast
CCN: Cellular communication network
CLEC11A: C-type lectin domain containing 11A
COL: Collagen
CSPG: Chondroitin-sulphate proteoglycan
CTSC: Cathepsin C
CXCL: Chemokine (C-X-C motif) ligand
DMFS: Distant metastasis-free survival
ECM: Extracellular matrix
ER: Oestrogen receptor
FBLN: Fibulin
FBN: Fibrillin
FGF: Fibroblasts growth factor
FFPE: Formalin-fixed paraffin-embedded
FN: Fibronectin
Fwd: Forward
GAG: Glycosaminoglycan
GPC1: Glypican-1
GOBO: Gene expression-based outcome for breast cancer online
HER2: Human epidermal growth factor receptor 2
HR: Hazard ratio
HSPG: Heparan sulphate proteoglycan
IGF1: Insulin-like growth factor 1
KRT: Keratin
LN: Lymph node
OMD: Osteomodulin
PAF: Pregnancy-associated fibroblast
POSTN: Periostin
Rev: Reverse
RFS: Recurrence-free survival
SFRP1: Secreted frizzled-related protein 1
SLIT2: Slit guidance ligand 2
TEB: Terminal end bud
TGFBI: Transforming growth factor beta-induced
TIMP1: Tissue inhibitor of matrix metalloproteinases 1
TNBC: Triple-negative breast cancer
TNC: Tenascin-C
VCAN: Versican
VEGFD: Vascular endothelial growth factor D
VIM: Vimentin
VTN: Vitronectin
WISP2: WNT1-inducible signaling pathway protein 2
WNT: Wingless and Int-1
