# Supplementary figures and images for: A matrisome RNA signature from early-pregnancy mouse mammary fibroblasts predicts distant metastasis-free breast cancer survival in humans
Source: Breast Cancer Res. 2021 Sep 26;23:90. doi: 10.1186/s13058-021-01470-3 (PMC8474794; doi:10.1186/s13058-021-01470-3)

Figure S1: Structural changes in the ECM during early pregnancy in the 3<sup>rd</sup> (thoracic) gland

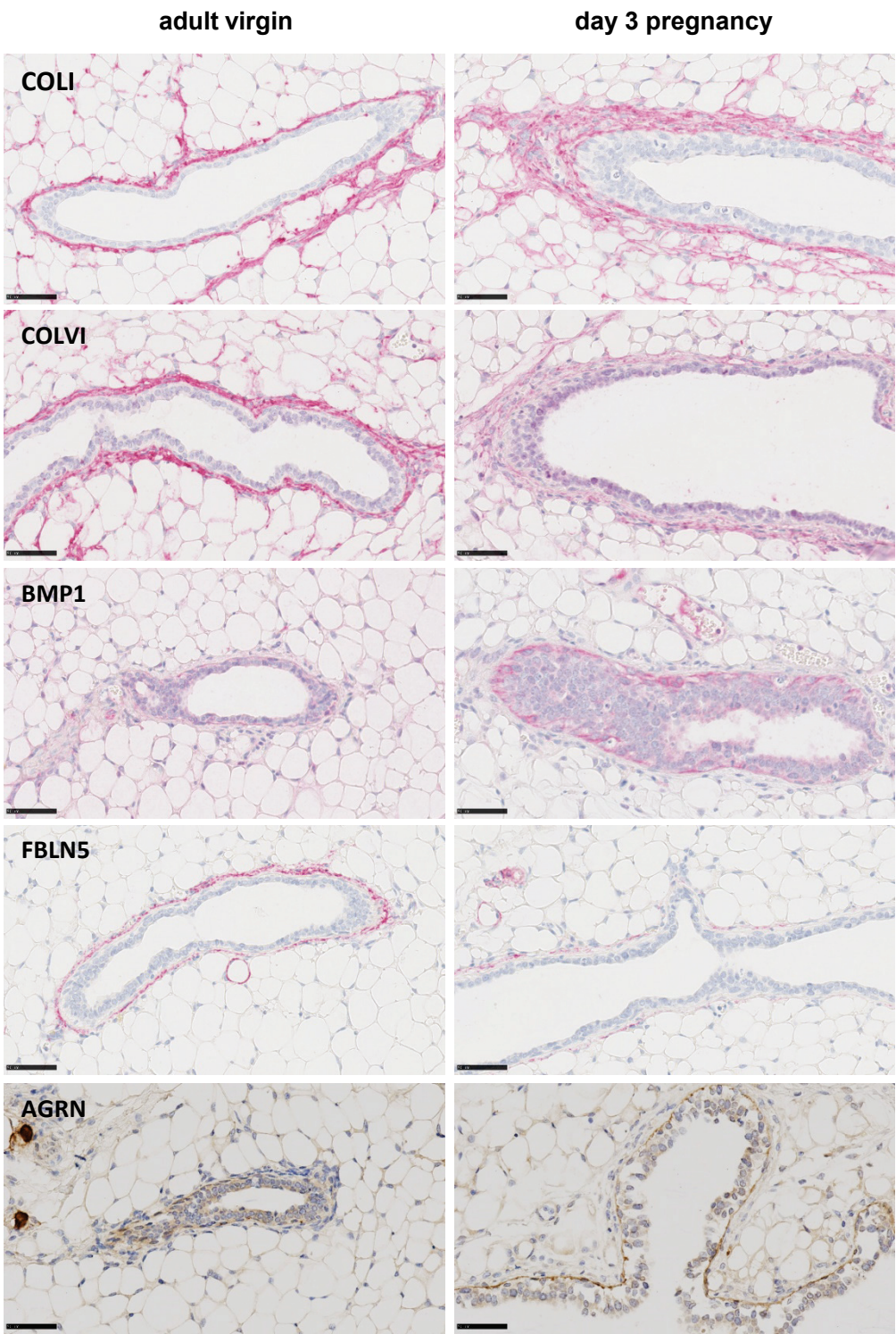

Supplement: Supplementary file 1 — Additional file 1: Figure S1. Immunohistochemical analysis of 3-days pregnant mouse (n = 1 animal, 3rd gland) mammary gland and age-matched adult virgin glands showing the changes of matrix- and basement membrane-associated proteins (COLI, COLVI, BMP1, FBLN5, and AGRN). Scale bars are 50 µm. [file 13058_2021_1470_MOESM1_ESM.pdf]

Figure S2: Confirmation of pregnancy by induced FBLN2 staining

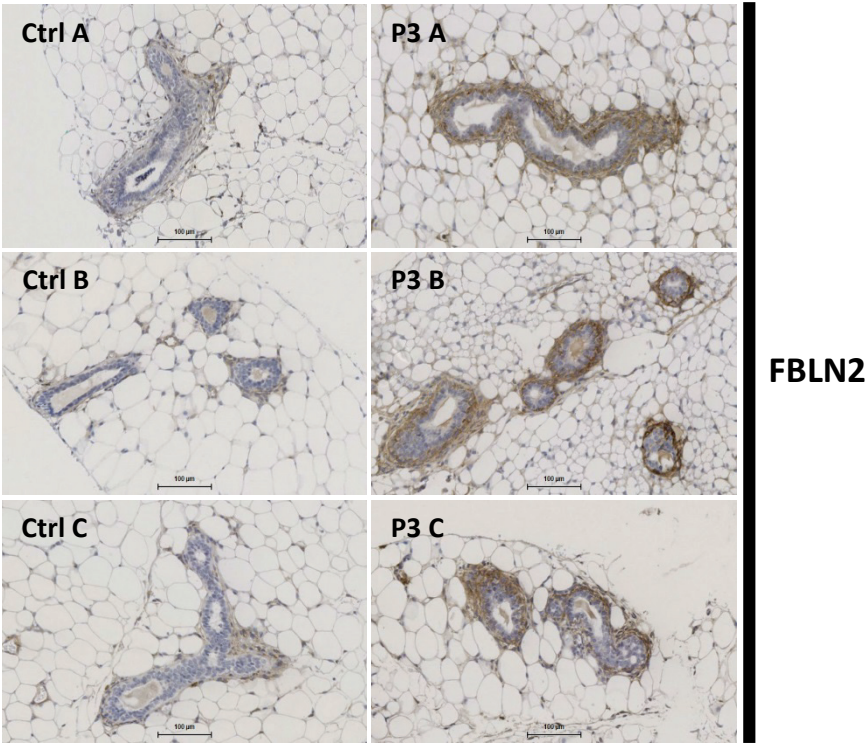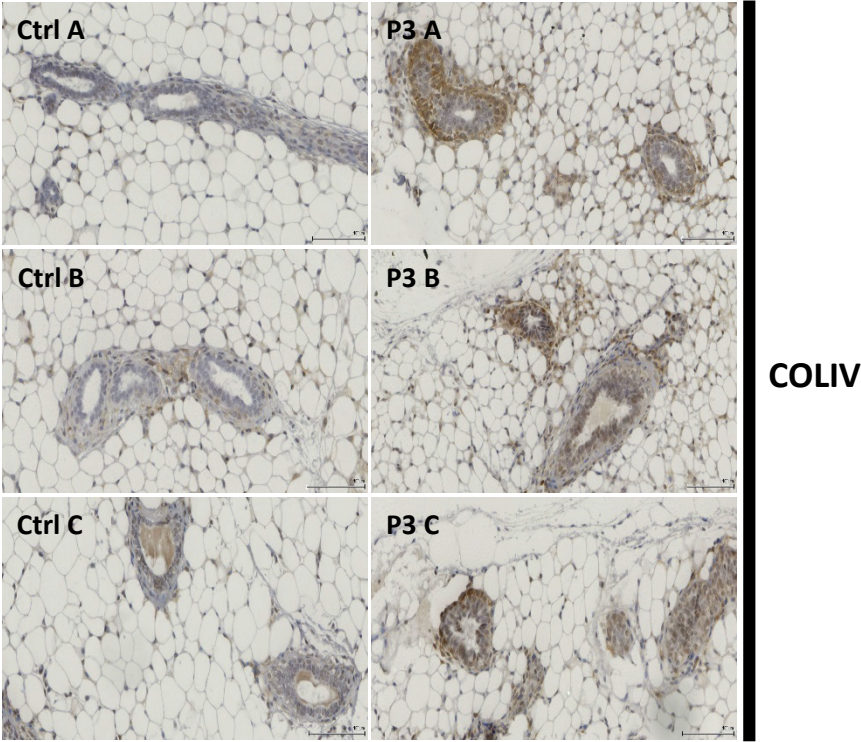

Supplement: Supplementary file 3 — Additional file 3: Figure S2. Immunohistochemical analysis using the contralateral inguinal mammary glands for validation of the specific expression of FBLN2 around the outgrowing ductal epithelium as a marker of early pregnancy in all three mice used for the isolation of 3-days pregnancy-associated fibroblasts (P3 A-C) and their age-matched control counterparts (Ctrl A-C). Similar results were obtained for COLIV. Scale bars are 100 µm. [file 13058_2021_1470_MOESM3_ESM.pdf]

Figure S3: Microarray results confirm fibroblast enrichment

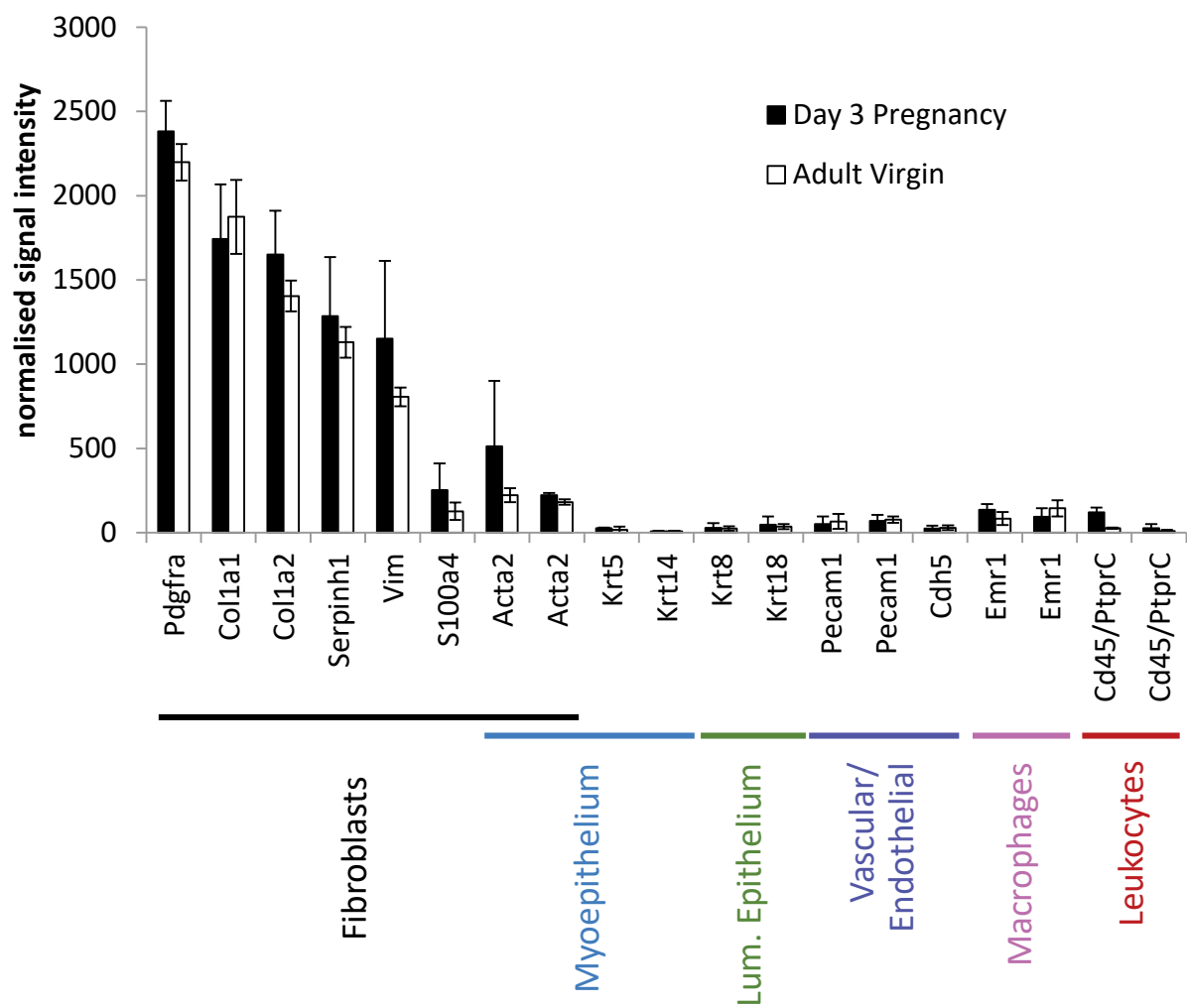

Supplement: Supplementary file 4 — Additional file 4: Figure S3. Microarray signal intensities of markers for fibroblasts (Pdgfra (probe_ID ILMN_1235932), Col1a1 (ILMN_2687872), Col1a2 (ILMN_1253806), Serpinh1 (Hsp47; ILMN_2822850), Vim (ILMN_2451022), S100a4 (ILMN_2731901)), fibroblast/myoepithelial cells (Acta2 (smooth-muscle actin; ILMN_2693895; ILMN_27103549)), myopepithelial cells (Krt5 (ILMN_2740939), Krt14 (ILMN_2722616)), luminal epithelial cells (Krt8 (ILMN_1221157), Krt18 (ILMN_2711267)), vascular/endothelial cells (Pecam1 (Cd31; ILMN_2700982; ILMN_3147074), Cdh5 (VE-cadherin)), macrophages (Emr1 (ILMN_1216880; ILMN_2847787)), as well as leukocytes (PtprC (Cd45; ILMN_1212836; ILMN_2671984)) from PAFs (Day3 Pregnancy) and from fibroblasts isolated from age-matched virgin counterparts (Adult Virgin). Bars represent standard errors (n = 3 animals). Identically labelled columns represent the signal intensities from individual probes for the same RNA. [file 13058_2021_1470_MOESM4_ESM.pdf]

Figure S4: IHC staining for VCAN and ALX4

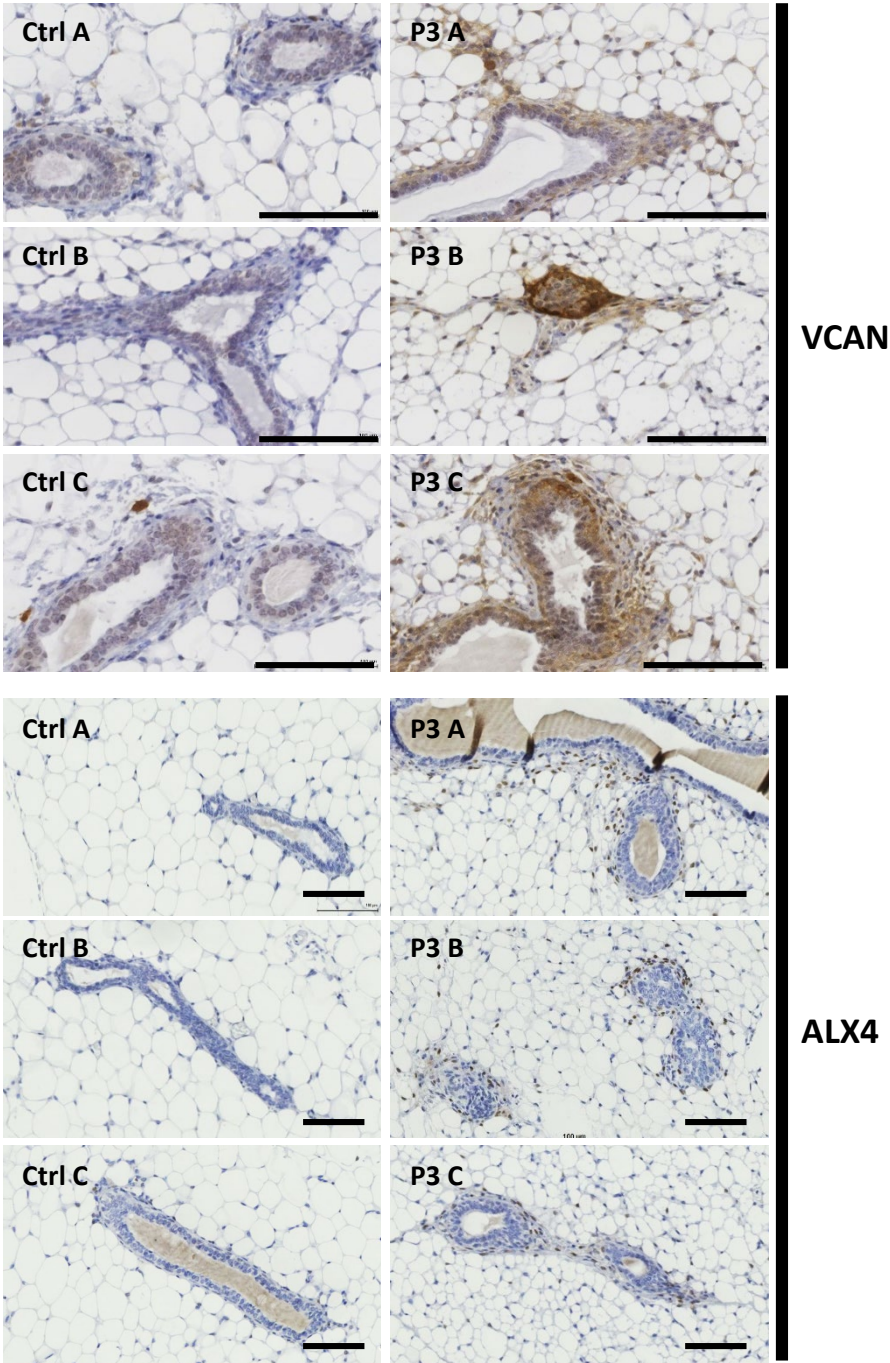

Supplement: Supplementary file 6 — Additional file 6: Figure S4. Immunohistochemical analysis using the contralateral inguinal mammary glands for validation of the specific expression of VCAN around the outgrowing ductal epithelium in the three mice used for the isolation of 3-days pregnancy-associated fibroblasts (P3 A-C) and their age-matched control counterparts (Ctrl A-C). Similar results were obtained for ALX4. Note that for ALX4 Ctrl A and P3 A show the same sections as in Fig. 3A. Scale bars are 100 µm. [file 13058_2021_1470_MOESM6_ESM.pdf]

Figure S6: KM analysis, using the 64-gene signature (DMFS)

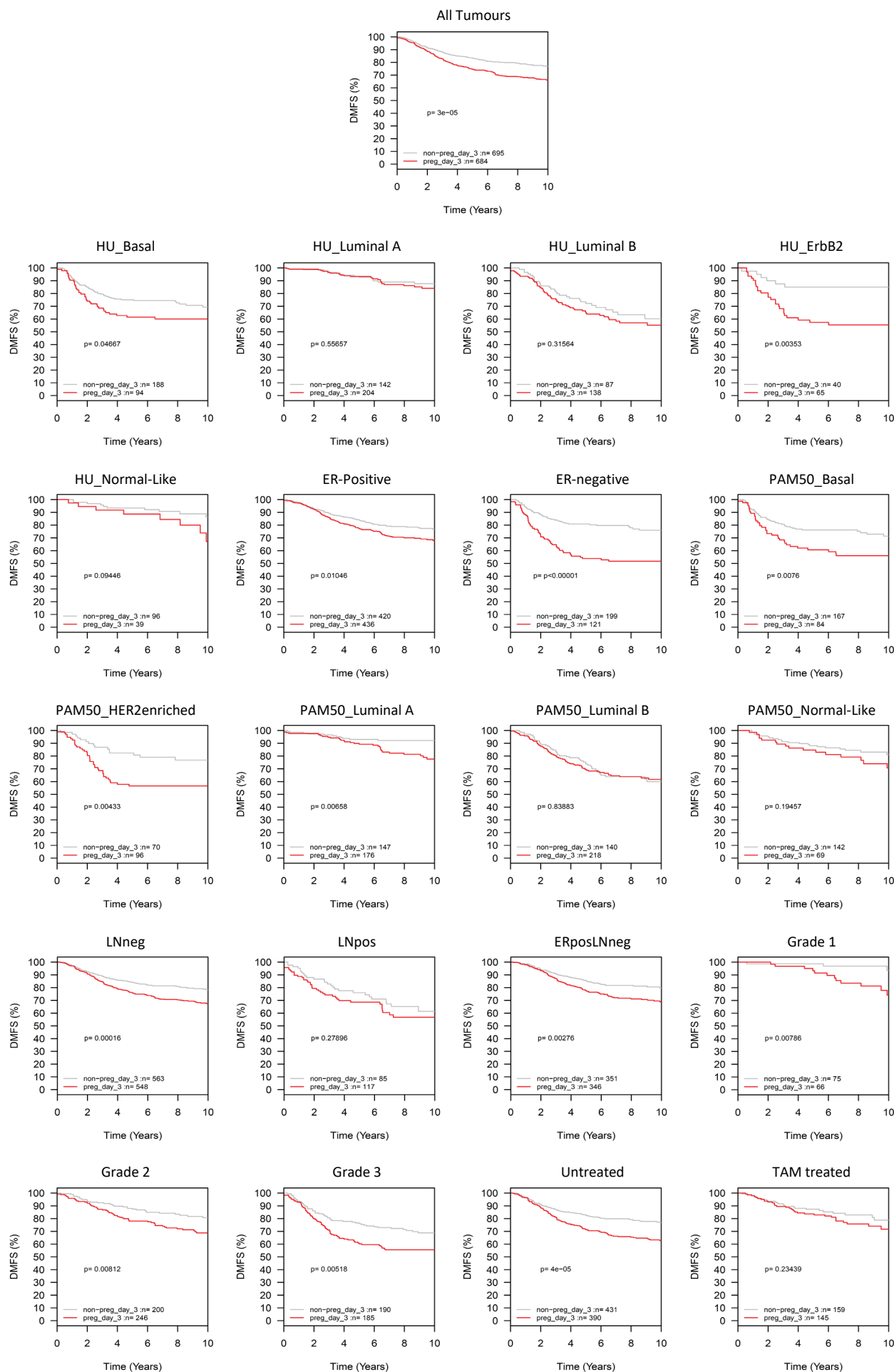

Supplement: Supplementary file 9 — Additional file 9: Figure S6. Kaplan–Meier analyses generated using the GOBO online tool for the 64-gene matrisome signature (DMFS) in all patients and each breast cancer subgroup classified based on ER status, Tumor grade, LN status and molecular subtyping according to Hu et al. [49–51]or PAM50 (Luminal A, Luminal B, Her-2, Basal and Normal-like) [52], as well as Tamoxifen treatment. [file 13058_2021_1470_MOESM9_ESM.pdf]

Figure S7: KM-analysis, using the 64-gene signature (RFS)

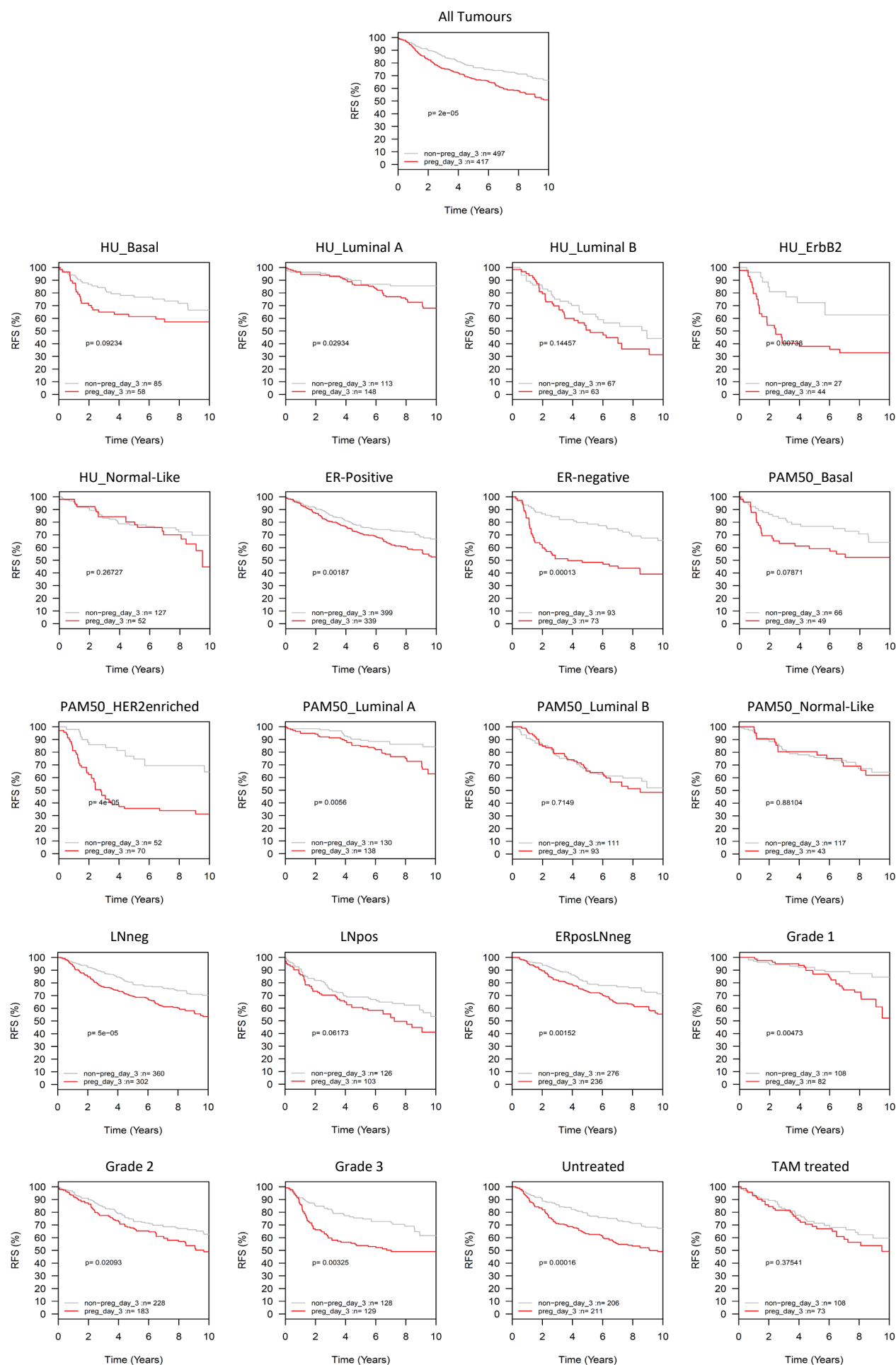

Supplement: Supplementary file 10 — Additional file 10: Figure S7. Kaplan–Meier analyses generated using the GOBO online tool for the 64-gene matrisome signature (RFS) in all patients and each breast cancer subgroup classified based on ER status, Tumor grade, LN status and molecular subtyping according to Hu et al. [49–51]or PAM50 (Luminal A, Luminal B, Her-2, Basal and Normal-like) [52], as well as Tamoxifen treatment. [file 13058_2021_1470_MOESM10_ESM.pdf]

Figure S9: Prognostic power of the 18 individual genes in total GOBO dataset - DMFS

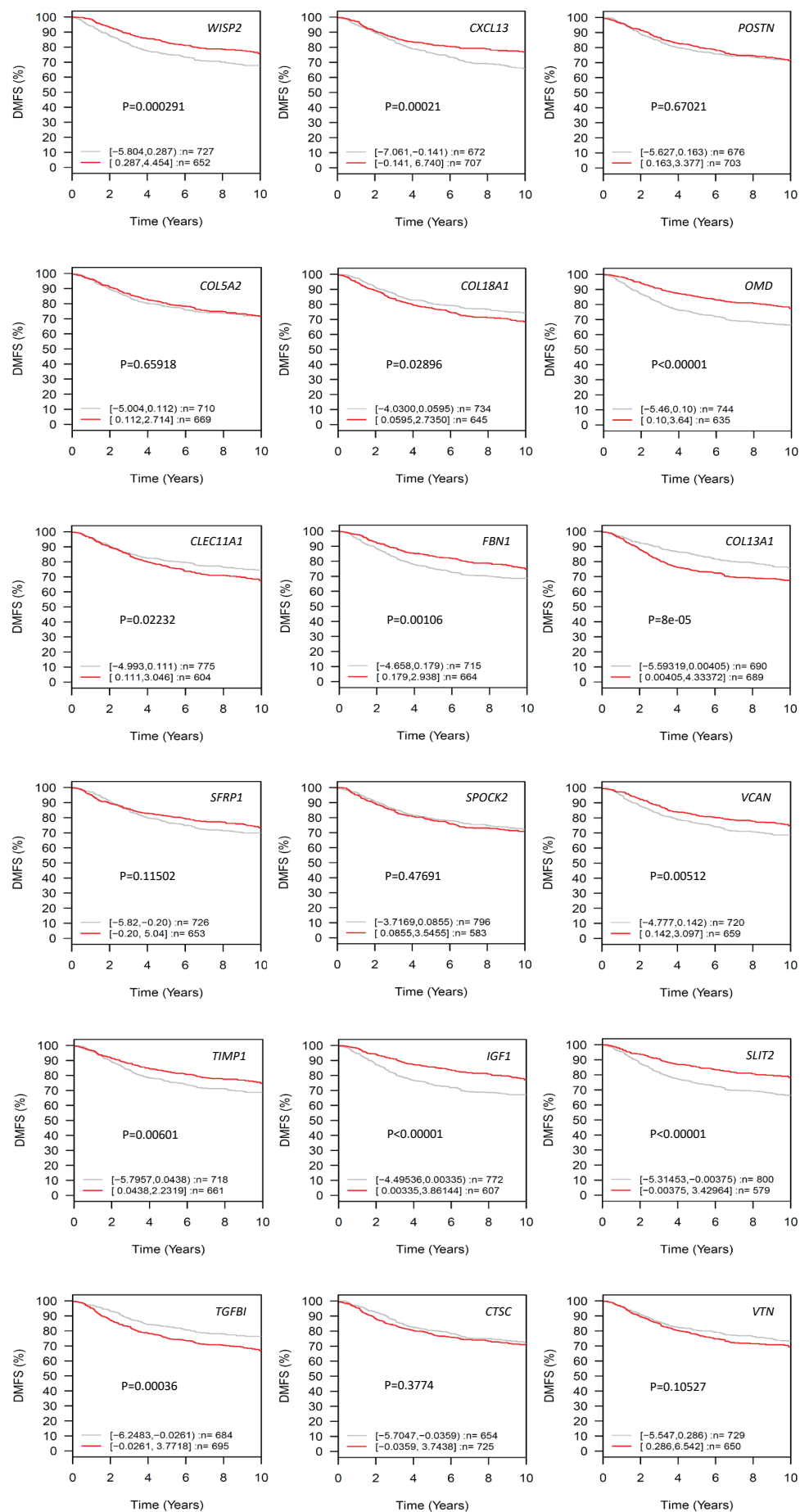

Supplement: Supplementary file 12 — Additional file 12: Figure S9. Kaplan–Meier analyses generated using the GOBO online tool for each single gene of the shortened 18-gene signature (DMFS) in the total breast cancer dataset (showing non-adjusted p-values). Cut-offs of median expression for each gene were automatically chosen by GOBO for best separation into two survival groups. [file 13058_2021_1470_MOESM12_ESM.pdf]

Figure S11: KM analysis, using the 18-gene signature (DMFS)

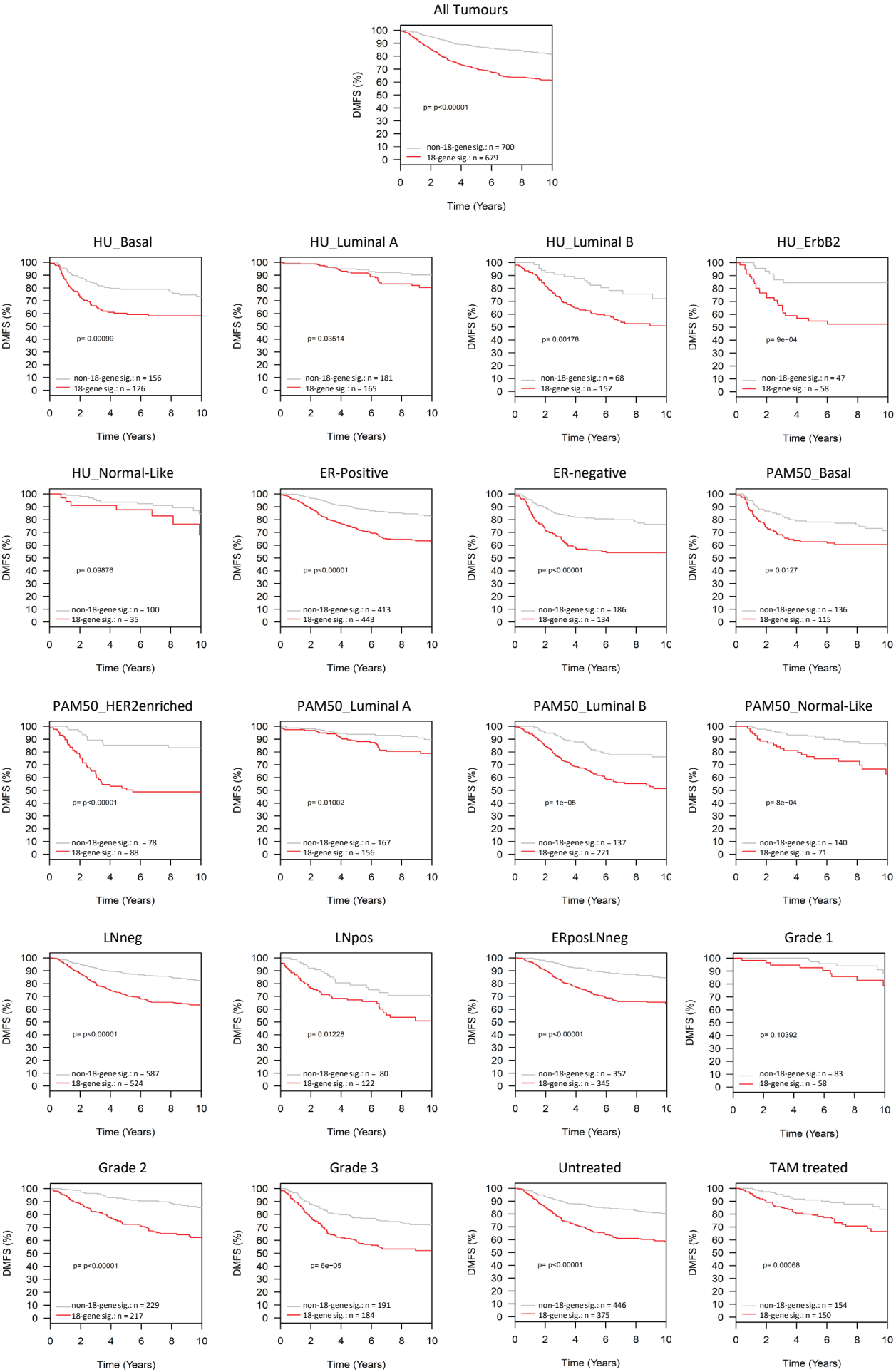

Supplement: Supplementary file 14 — Additional file 14: Figure S11. Kaplan–Meier analyses generated using the GOBO online tool for the shortened 18-gene matrisome signature (DMFS) in all patients and each breast cancer subgroup classified based on ER status, Tumor grade, LN status and molecular subtyping according to Hu et al. [49–51]or PAM50 (Luminal A, Luminal B, Her-2, Basal and Normal-like) [52], as well as Tamoxifen treatment. [file 13058_2021_1470_MOESM14_ESM.pdf]

Figure S12: KM analysis, using the 18-gene signature (RFS)

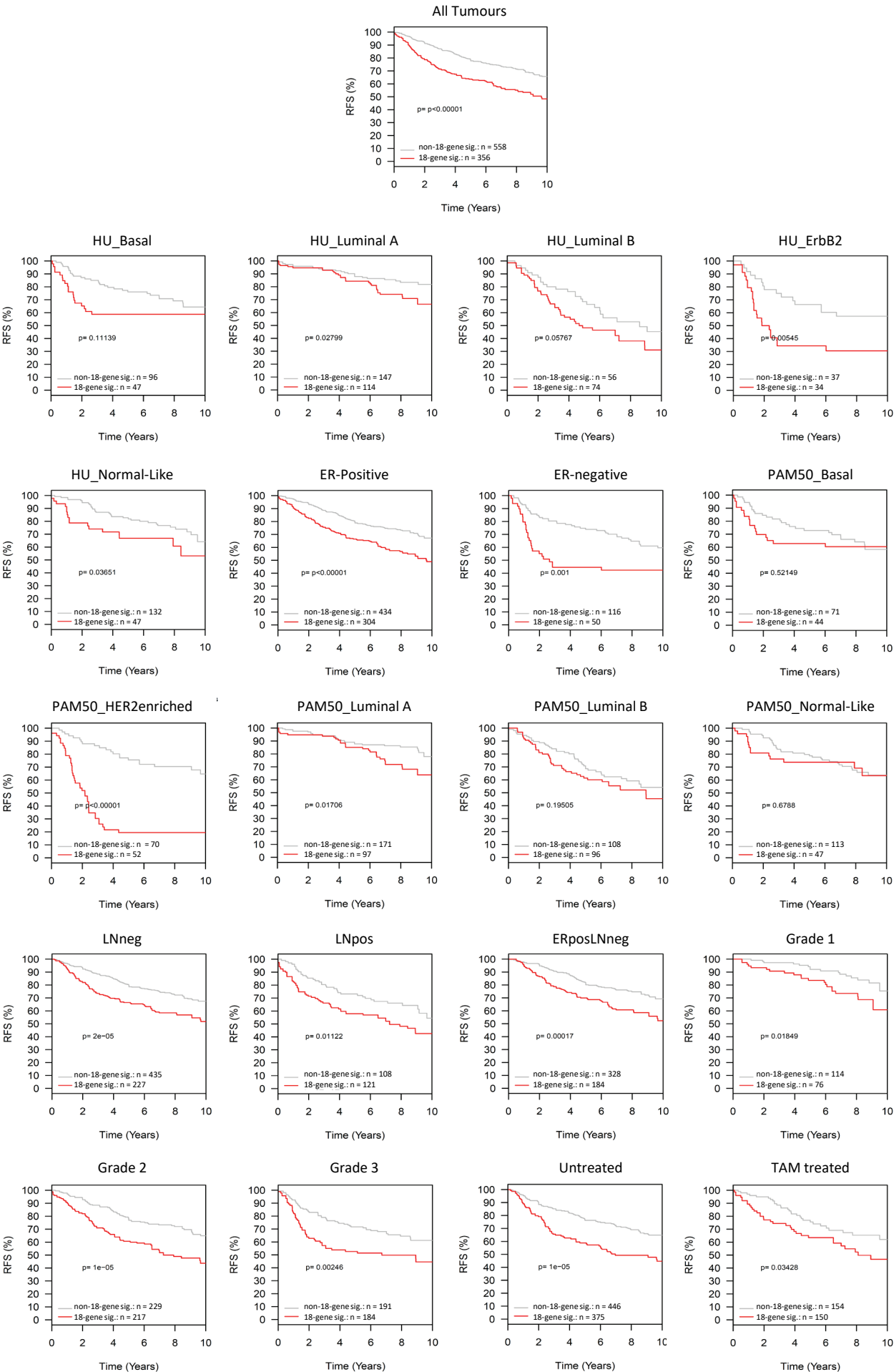

Supplement: Supplementary file 15 — Additional file 15: Figure S12. Kaplan–Meier analyses generated using the GOBO online tool for the shortened 18-gene matrisome signature (RFS) in all patients and each breast cancer subgroup classified based on ER status, Tumor grade, LN status and molecular subtyping according to Hu et al. [49–51] or PAM50 (Luminal A, Luminal B, Her-2, Basal and Normal-like) [52], as well as Tamoxifen treatment. [file 13058_2021_1470_MOESM15_ESM.pdf]
