# Supplementary material for: A matrisome RNA signature from early-pregnancy mouse mammary fibroblasts predicts distant metastasis-free breast cancer survival in humans
Source: Breast Cancer Res. 2021 Sep 26;23:90. doi: 10.1186/s13058-021-01470-3 (PMC8474794; doi:10.1186/s13058-021-01470-3)
Supplement: Supplementary file 2 — Additional file 2: Table S1. Table with total and average counts (and standard deviation) of identified 3D-structures from triplicate co-culture experiments. Student T-tests were performed to compare co-cultures with fibroblasts from P3 and age-matched control (V12) mice. [file 13058_2021_1470_MOESM2_ESM.pdf]

Structures counted in 4 random fields per well (in triplicate)

Structure	Control (no fibroblasts)			with V12 fibroblasts			with P3 fibroblasts		
small spheriods (rounded without branching)	92	91	93	53	50	56	48	50	48
small branched spheriods	0	0	0	28	21	23	20	27	26
Large spheriods (rounded without branching)	8	9	7	7	12.5	10	8	5	9
Large branched spheriods	0	0	0	10	16.5	7	12	10	5
Large with highly branched spheriods	0	0	0	2	0	3	12	10	12

	**Total Count**			**Average**			**Standard Deviation**			**T-Test**
	Ctrl	V12	P3	Ctrl	V12	P3	Ctrl	V12	P3	p-value (V12 vs P3)
**small spheroids**	276	159	146	92	53	48.667	1	3	1.1547	0.116
**small branched**	0	72	73	0	24	24.333	0	3.60555	3.7859	0.917
**large spheroids**	24	29.5	22	8	9.8333	7.3333	1	2.75379	2.0817	0.283
**large branched**	0	33.5	27	0	11.167	9	0	4.85627	3.6056	0.571
**large highly branched**	0	5	34	0	1.6667	11.333	0	1.52753	1.1547	**0.001**
