# Supplementary material for: A matrisome RNA signature from early-pregnancy mouse mammary fibroblasts predicts distant metastasis-free breast cancer survival in humans
Source: Breast Cancer Res. 2021 Sep 26;23:90. doi: 10.1186/s13058-021-01470-3 (PMC8474794; doi:10.1186/s13058-021-01470-3)
Supplement: Supplementary file 7 — Additional file 7: Figure S5. STRING analysis using the 64-gene matrisome genes showing their predicted protein–protein interactions. The table below shows the two most significantly over-represented Gene Ontology (GO) biological processes, molecular functions, and local network clusters with enrichment-level (strength) and false-discovery rate (FDR) for each grouping. The ‘Count in gene set’ numbers represent the number of genes within our signature associated with a particular GO group out of the total of genes associated with this group. [file 13058_2021_1470_MOESM7_ESM.pdf]

Figure S5: STRING Analysis of 64 identified Matrisome genes

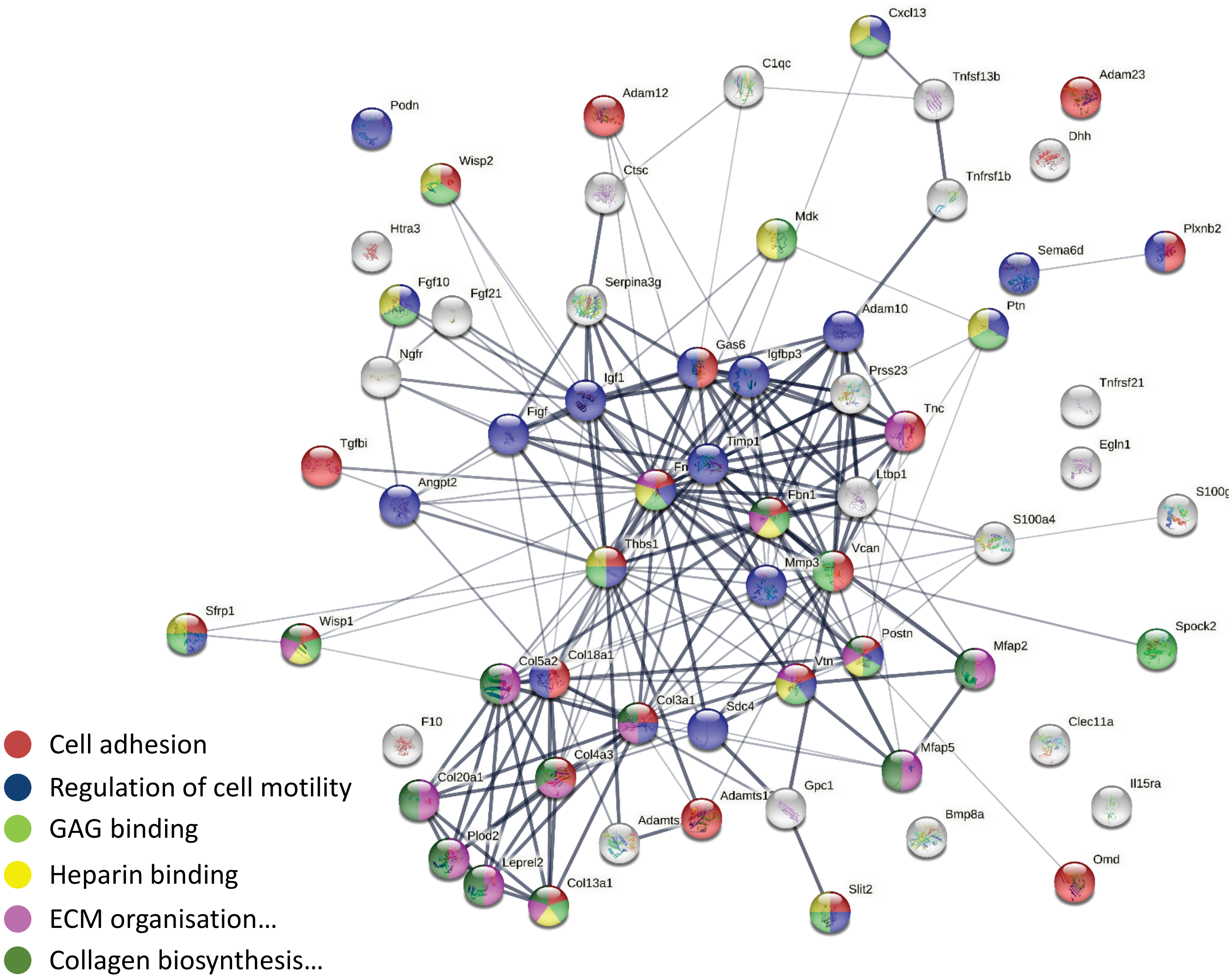

GO-term	Description	Count in gene set	Strength	FDR
**Biological Processes**				
GO:0030198	Cell adhesion	22 of 705	1.03	8.69e-14
GO:0030334	Regulation of cell migration	23 of 805	0.99	8.69e-14
**Molecular Functions**				
GO:0005539	Glycosaminoglycan binding	16 of 184	1.48	8.50e-17
GO:0008201	Heparin binding	14 of 139	1.54	1.30e-15
**Local Network Cluster**				
CL:21858	ECM organisation, and Focal adhesion	15 of 156	1.52	2.95e-16
CL:21860	Collagen biosynthesis and modifying enzymes, and Elastic fibre formation	12 of 181	1.71	3.05e-15
