# Supplementary material for: A matrisome RNA signature from early-pregnancy mouse mammary fibroblasts predicts distant metastasis-free breast cancer survival in humans
Source: Breast Cancer Res. 2021 Sep 26;23:90. doi: 10.1186/s13058-021-01470-3 (PMC8474794; doi:10.1186/s13058-021-01470-3)

# Figure S8: Adjusted LogRank p-values for 18 genes with significant difference in at least one breast cancer subgroup

A

| Gene | WISP2 | CXCL13 | POSTN | COL5A2 | COL18A1 | OMD | CLEC11A | FBN1 | COL13A1 | SFRP1 | SPOCK2 | *VCAN* | *TIMP1* | *IGF1* | *SLIT2* | *TGFBI* | *CTSC* | *VTN* |
|---|---|---|---|---|---|---|---|---|---|---|---|---|---|---|---|---|---|---|
| **ALL** | 0.0103 | 0.0084 | 0.8886 | 0.8882 | 0.1796 | 0.0004 | 0.1528 | 0.0264 | 0.0039 | 0.3818 | 0.7841 | 0.0698 | 0.0753 | 0.0004 | 0.0004 | 0.0119 | 0.7220 | 0.3702 |
| **Basal** | 0.7477 | 0.0806 | 0.2127 | 0.4836 | 0.6456 | 0.5207 | 0.1850 | 0.8882 | 0.4503 | 0.8998 | 0.8172 | 0.2164 | 0.8665 | 0.3702 | 0.8612 | 0.1768 | 0.0058 | 0.8923 |
| **Luminal_A** | 0.3818 | 0.1591 | 0.3521 | 0.3521 | 0.2407 | 0.0596 | 0.4777 | 0.3602 | 0.2804 | 0.7770 | 0.1363 | 0.0475 | 0.3521 | 0.3818 | 0.2885 | 0.8612 | 0.3820 | 0.9582 |
| **Luminal_B** | 0.0774 | 0.6905 | 0.8218 | 0.8407 | 0.0872 | 0.2490 | 0.2778 | 0.9255 | 0.1079 | 0.9419 | 0.8803 | 0.8998 | 0.8218 | 0.1850 | 0.8998 | 0.7410 | 0.7528 | 0.8612 |
| **Her2** | 0.7122 | 0.0027 | 0.2778 | 0.0264 | 0.2639 | 1.0000 | 0.6224 | 0.8612 | 1.0000 | 0.6762 | 0.0731 | 0.8527 | 1.0000 | 0.9226 | 0.4275 | 0.7470 | 0.0936 | 0.7514 |
| **Normal-like** | 0.9842 | 0.5869 | 0.7863 | 1.0000 | 0.7503 | 0.6519 | 0.9194 | 0.9132 | 0.4026 | 1.0000 | 0.9591 | 0.4836 | 0.7672 | 1.0000 | 0.9941 | 0.9941 | 1.0000 | 0.6661 |
| **ERpos** | 0.0979 | 0.2639 | 0.5390 | 0.4275 | 0.3405 | 0.0027 | 0.0768 | 0.2785 | 0.0092 | 0.0495 | 0.3702 | 0.0451 | 0.1312 | 0.0010 | 0.0004 | 0.1129 | 0.6037 | 0.0451 |
| **ERneg** | 0.8612 | 0.0006 | 0.1102 | 0.1045 | 0.0700 | 0.7528 | 0.1521 | 0.8638 | 0.2722 | 0.8612 | 0.4554 | 0.5207 | 0.4780 | 0.9582 | 0.8231 | 0.0173 | 0.0995 | 0.5515 |
| **PAM50_Basal** | 0.8886 | 0.0806 | 0.2550 | 0.5159 | 0.5763 | 0.9579 | 0.1110 | 0.7122 | 0.3702 | 0.9758 | 0.7510 | 0.2708 | 1.0000 | 0.3105 | 0.6639 | 0.1079 | 0.0137 | 0.7470 |
| **PAM50_ Her2** | 0.5059 | 0.0058 | 0.3442 | 0.1528 | 0.1178 | 0.6221 | 0.2341 | 0.7122 | 0.7916 | 0.9779 | 0.0057 | 0.8886 | 0.9888 | 0.9591 | 0.2163 | 0.7642 | 0.1453 | 1.0000 |
| **PAM50_Lum_A** | 0.5207 | 0.6291 | 0.9742 | 0.9730 | 0.1079 | 0.3521 | 0.6492 | 0.5763 | 0.1044 | 0.4934 | 1.0000 | 0.3530 | 0.7925 | 0.1508 | 0.0326 | 0.0872 | 0.8612 | 0.5231 |
| **PAM50_Lum_B** | 0.3702 | 0.0698 | 0.6739 | 0.9842 | 0.3225 | 0.0745 | 0.3521 | 0.5346 | 0.0184 | 0.9779 | 0.7122 | 0.5349 | 0.7294 | 0.4295 | 0.8998 | 0.4835 | 0.5763 | 0.2240 |
| **PAM50_Norm** | 0.4339 | 0.3818 | 0.8853 | 0.8612 | 0.8337 | 0.3702 | 0.6739 | 0.9766 | 0.7335 | 1.0000 | 0.2042 | 0.4980 | 0.2245 | 0.3654 | 0.4682 | 0.9108 | 0.4503 | 0.2006 |
| **LN_neg** | 0.0495 | 0.0234 | 0.7470 | 0.8530 | 0.3554 | 0.0011 | 0.1102 | 0.0393 | 0.0145 | 0.3702 | 0.6636 | 0.0393 | 0.0092 | 0.0016 | 0.0004 | 0.0451 | 0.5763 | 0.3521 |
| **LN_pos** | 0.6456 | 0.8218 | 0.7679 | 0.9941 | 0.8064 | 0.3521 | 0.6896 | 0.6907 | 0.3818 | 0.9941 | 0.8730 | 0.9001 | 0.6401 | 0.4836 | 0.8391 | 0.2681 | 0.6987 | 0.3530 |
| **ERpos_LNeg** | 0.1268 | 0.2520 | 0.4984 | 0.3521 | 0.2773 | 0.0367 | 0.0482 | 0.2488 | 0.0204 | 0.0682 | 0.4617 | 0.0376 | 0.0495 | 0.0032 | 0.0004 | 0.2006 | 0.7528 | 0.0939 |
| **Grade_1** | 0.4835 | 0.7565 | 0.4835 | 0.8696 | 0.8998 | 0.0662 | 0.8026 | 0.2550 | 0.3611 | 0.7528 | 0.6221 | 0.4556 | 0.6762 | 0.0745 | 0.6456 | 0.7528 | 0.8407 | 0.8796 |
| **Grade_2** | 0.1199 | 0.0111 | 0.7672 | 0.7021 | 0.7657 | 0.0037 | 0.9266 | 0.0662 | 0.2319 | 0.7614 | 0.8612 | 0.1508 | 0.5869 | 0.1074 | 0.2717 | 0.8998 | 0.3583 | 0.3993 |
| **Grade_3** | 0.8886 | 0.0145 | 0.0495 | 0.0835 | 0.0305 | 0.9551 | 0.1045 | 0.6456 | 0.6762 | 0.5810 | 0.9842 | 0.8886 | 0.9941 | 0.5189 | 0.4893 | 0.1643 | 0.1690 | 0.6218 |
| **untreated** | 0.0601 | 0.0084 | 0.8612 | 0.9579 | 0.4270 | 0.0039 | 0.5517 | 0.0975 | 0.0326 | 0.5515 | 1.0000 | 0.0774 | 0.0367 | 0.0010 | 0.0010 | 0.1095 | 0.8612 | 0.7357 |
| **TAM-treated** | 0.5994 | 0.9226 | 0.0872 | 0.7122 | 0.8886 | 0.0444 | 0.2042 | 0.1209 | 0.4556 | 0.1102 | 0.7021 | 0.1045 | 0.9344 | 0.0596 | 0.3521 | 0.9369 | 0.5174 | 0.1435 |

*LogRank p-values adjusted using Benjamini-Hochberg multiple testing correction

**Expression associated with poor DMFS:** **= High** **= Low**

B

| Gene Symbol | Fold-Change Preg vs Ctrl |
|---|---|
| ***Wisp2*** | -3.31 |
| ***Cxcl13*** | -2.70 |
| ***Postn**** | 1.79 |
| ***Omd*** | -1.93 |
| ***Col5a2*** | 2.29 |
| ***Col18a1*** | 2.71 |
| ***Fbn1*** | -2.63 |
| ***Col13a1*** | 2.21 |
| ***Sfrp1*** | -2.21 |
| ***Spock2*** | -1.74 |
| ***Clec11a/Scgf*** | 1.17 |
| ***Vcan*** | 3.10 |
| ***Timp1**** | 1.75 |
| ***Igf1*** | 1.43 |
| ***Slit2*** | 2.02 |
| ***Tgfbi*** | -1.73 |
| ***Ctsc*** | 1.94 |
| ***Vtn*** | -1.48 |

11_gene_sig (Wisp2 through Clec11a/Scgf)

7_gene_sig (Vcan through Vtn)

*average of multiple probes*

C

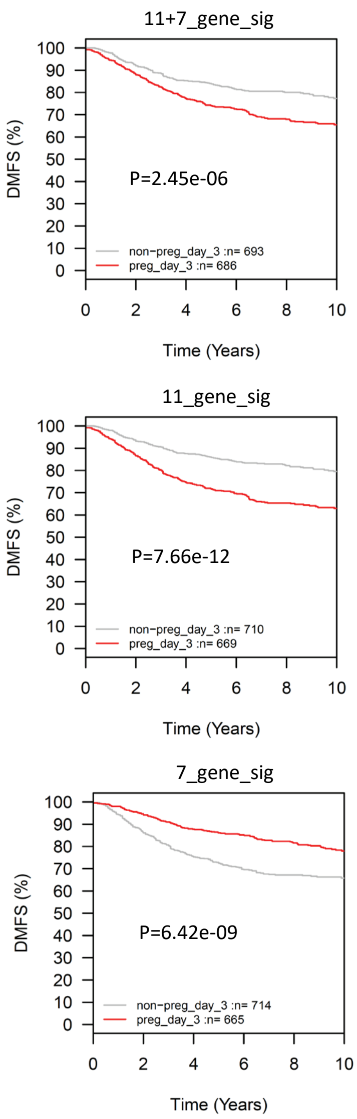

Supplement: Supplementary file 11 — Additional file 11: Figure S8. (A) List of the multiple-correction adjusted p-values for those genes with a p-value of p < 0.05 in at least one breast cancer subtype. Colours show the association of either high (red) or low (blue) expression for each gene that is associated with poor DMFS. (B) List of the same RNAs with fold-change expression values between PAFs (Preg) and control (Ctrl) fibroblasts. The average fold-change of multiple probes was used for Postn and Timp1 (*). (C) Kaplan–Meier analysis for gene signatures of either those 11 genes for which the expression (up or down) during pregnancy is also associated with poor DMFS in breast cancer (11_gene_sig; WISP2, CXCL13, POSTN, COL5A2, COL13A1, COL18A1, OMD, CLEC11A, FBN1, SFRP1, SPOCK2), and the seven genes for which the expression (up or down) during pregnancy is associated with improved DMFS (7_gene_sig; VCAN, TIMP1, IGF1, SLIT2, TGFBI, CTSC, VTN), or a combination of the two (11 + 7_gene_sig). [file 13058_2021_1470_MOESM11_ESM.pdf]
