# Supplementary material for: A matrisome RNA signature from early-pregnancy mouse mammary fibroblasts predicts distant metastasis-free breast cancer survival in humans
Source: Breast Cancer Res. 2021 Sep 26;23:90. doi: 10.1186/s13058-021-01470-3 (PMC8474794; doi:10.1186/s13058-021-01470-3)
Supplement: Supplementary file 13 — Additional file 13: Figure S10. Multivariate Analysis for 18-gene signature (18_gene_sig) in the total breast cancer cohort (Total BC cohort), ERpos (ER_pos) or ERneg (ER_neg) cancer patients in the presence of other clinical parameters: size > 20 mm (Size), age > 50 years (Age), grade (G3), LN-status (LNneg) and ER-status (ERpos), using either DMFS or RFS as endpoint (10-year cut-off). [file 13058_2021_1470_MOESM13_ESM.pdf]

Figure S10: Multivariate Analysis for the 18-gene signature

Multivariate Analysis -DMFS

Total BC cohort	p-value	Hazard Ratio
18-gene sig	5.61E-09	2.42 (1.8-3.26)
ER_pos	0.038	0.71 (0.51-0.98)
LNneg	0.048	0.7 (0.49-1)
Grade:3	3.00E-04	1.75 (1.3-2.37)
Age: >50 years	0.648	1.07 (0.8-1.44)
Size: >20mm	0.077	1.3 (0.97-1.74)

ER_pos	p-value	Hazard Ratio
18-gene sig	5.87E-07	2.61 (1.79-3.81)
ER_pos	NA	NA
LNneg	0.228	0.77 (0.5-1.18)
Grade:3	7.60E-05	2 (1.42-2.81)
Age: >50 years	0.794	0.95 (0.66-1.37)
Size: >20mm	0.014	1.58 (1.1-2.27)

ER_neg	p-value	Hazard Ratio
18-gene sig	0.006	2 (1.22-3.27)
ER_pos	NA	NA
LNneg	0.079	0.57 (0.3-1.07)
Grade:3	0.674	1.13 (0.64-1.99)
Age: >50 years	0.251	1.33 (0.82-2.18)
Size: >20mm	0.887	0.96 (0.59-1.59)

Multivariate Analysis -RFS

Total BC cohort	p-value	Hazard Ratio
18-gene sig	3.08E-08	1.98 (1.55-2.52)
ER_pos	0.026	0.7 (0.51-0.96)
LNneg	0.022	0.73 (0.56-0.96)
Grade:3	0.492	1.1 (0.83-1.45)
Age: >50 years	0.027	0.75 (0.58-0.97)
Size: >20mm	1.23E-05	1.73 (1.35-2.21)

ER_pos	p-value	Hazard Ratio
18-gene sig	8.14E-06	1.86 (1.42-2.44)
ER_pos	NA	NA
LNneg	0.086	0.77 (0.58-1.04)
Grade:3	0.183	1.23 (0.91-1.68)
Age: >50 years	0.043	0.74 (0.55-0.99)
Size: >20mm	1.89E-06	1.97 (1.49-2.61)

ER_neg	p-value	Hazard Ratio
18-gene sig	6.50E-04	2.42 (1.46-4.02)
ER_pos	NA	NA
LNneg	0.084	0.55 (0.28-1.08)
Grade:3	0.672	0.89 (0.52-1.52)
Age: >50 years	0.185	0.7 (0.41-1.19)
Size: >20mm	0.625	1.14 (0.68-1.89)
