# Supplementary material for: A matrisome RNA signature from early-pregnancy mouse mammary fibroblasts predicts distant metastasis-free breast cancer survival in humans
Source: Breast Cancer Res. 2021 Sep 26;23:90. doi: 10.1186/s13058-021-01470-3 (PMC8474794; doi:10.1186/s13058-021-01470-3)
Supplement: Supplementary file 16 — Additional file 16: Table S4. Comparison of the 18-gene signature (18_gene_sig) with the 21 gene signature from OncotypeDX and the 8 gene signature from Endopredict, using ‘Gene Set Analysis’ tool in GOBO, which allowed for analysis of weighted expression as previously defined [46, 48]. DMFS was chosen as endpoint (10-year cut-off). p-values for Kaplan–Meier analysis are shown for the total cohort and each breast cancer subgroup. Yellow highlights subgroups for which only the 18-gene signature shows a significant stratification (p < 0.05), while green highlights those subgroups, in which the 18-gene signature showed significance with the lowest p-value. [file 13058_2021_1470_MOESM16_ESM.docx]

**Supplementary Table S4**

| **Subset of Breast Tumours** | **18_gene_sig** | **Oncotype_DX** | **Endopredict** |
| --- | --- | --- | --- |
| **All** | 1.22E-15 | 9.99E-16 | 2.22E-15 |
| **HU_Basal** | 0.01605317 | 1 | 1 |
| **HU_Luminal A** | 0.001295012 | 5.75E-06 | 0.004573244 |
| **HU_Luminal B** | 0.084177903 | 0.082647631 | 0.003885612 |
| **HU_ERBB2** | 0.005508411 | 1 | 1 |
| **HU_Normal-like** | 0.008859737 | 0.577639412 | 0.352849824 |
| **ER-positive** | 1.85E-09 | 9.38E-13 | 1.95E-10 |
| **ER-negative** | 5.39E-05 | 0.072820828 | 0.109002648 |
| **PAM50_Basal** | 0.057685248 | 1 | 1 |
| **PAM50_HER2enriched** | 0.000186407 | 1 | 1 |
| **PAM50_Luminal A** | 0.000881522 | 0.000153504 | 0.000652641 |
| **PAM50_Luminal B** | 0.000519427 | 0.001495989 | 0.001548462 |
| **PAM50_Normal-like** | 0.000133443 | 0.00648564 | 0.056254455 |
| **LNneg** | 1.63E-11 | 2.44E-15 | 5.83E-14 |
| **LNpos** | 0.005626264 | 0.006351143 | 0.025640212 |
| **ERposLNneg** | 1.18E-07 | 9.59E-12 | 5.96E-09 |
| **ERnegLNneg** | 1 | 1 | 1 |
| **Grade1** | 0.260636171 | 0.001474845 | 0.097677033 |
| **Grade2** | 1.04E-08 | 1.81E-07 | 1.20E-07 |
| **Grade3** | 0.002960564 | 0.110097181 | 0.117462025 |
| **Untreated** | 6.79E-13 | 8.12E-12 | 6.23E-09 |
| **TAM** | 0.008085902 | 0.000125829 | 2.41E-07 |
